# Digital Therapeutics in China: Comprehensive Review

**DOI:** 10.2196/70955

**Published:** 2025-05-27

**Authors:** Nan Jiang, Xiru Yu, Yuxi Yang, Guanqiao Li, Chanchan He, Man Ping Wang, Yih Chung Tham, Tien Yin Wong, Keqin Rao

**Affiliations:** 1 School of Healthcare Management Tsinghua University Beijing China; 2 Beijing Tsinghua Changgung Hospital Tsinghua University Beijing China; 3 Institute for Hospital Management Tsinghua University Beijing China; 4 Bloomberg School of Public Health Johns Hopkins University Baltimore, MD United States; 5 School of Basic Medical Sciences Tsinghua University Beijing China; 6 School of Biomedical Engineering Tsinghua University Beijing China; 7 Shanghai Institute of Medical Quality Shanghai China; 8 National Medical Products Administration Beijing China; 9 Vanke School of Public Health Tsinghua University Beijing China; 10 School of Nursing University of Hong Kong Hong Kong China (Hong Kong); 11 Department of Ophthalmology Yong Loo Lin School of Medicine National University of Singapore Singapore Singapore; 12 Centre for Innovation and Precision Eye Health Yong Loo Lin School of Medicine National University of Singapore Singapore Singapore; 13 Singapore Eye Research Institute Singapore National Eye Centre Singapore Singapore; 14 Tsinghua Medicine Tsinghua University Beijing China

**Keywords:** digital therapeutics, China, global experience, digital interventions, digital health

## Abstract

**Background:**

Digital therapeutics (DTx) are software-driven interventions that provide personalized, evidence-based treatments for various medical conditions. China’s rapid technological adoption, large population, and supportive government policies position it as a potential global leader in DTx. However, challenges remain in clinical trial standardization, regulatory approval, product development, and reimbursement models. A comprehensive assessment of clinical evidence, commercialization trends, and regulatory frameworks is essential for understanding China’s evolving DTx ecosystem and its global implications.

**Objective:**

This study systematically reviews and analyzes the DTx landscape in China, focusing on clinical trials, commercial products, regulatory frameworks, and pricing and reimbursement models. The findings provide insights for countries aiming to develop, regulate, and integrate DTx solutions into health care systems.

**Methods:**

This comprehensive review integrates multiple methodological approaches to examine different aspects of the DTx ecosystem in China. We conducted a systematic review and meta-analysis to evaluate clinical trials, searching PubMed, Google Scholar, IEEE, Web of Science, and ScienceDirect until July 2024. Meta-analyses used random-effects models, reporting results as standardized mean differences (SMDs) and 95% CIs. For commercial products, a scoping review using the National Medical Products Administration database was performed. Regulatory policies were systematically identified through a manual review of official government sources, while pricing and reimbursement models were analyzed through a comprehensive assessment of public and private insurance policies, government initiatives, and commercial pathways.

**Results:**

**:** A total of 96 clinical trials on DTx in China were identified, with cognitive disorders (n=21, 22%) and diabetes (n=20, 21%) being the most frequently studied, followed by cardiovascular diseases (n=8, 8%), sleeping disorders (n=6, 6%), and smoking cessation (n=6, 6%). Meta-analysis for diabetes DTx showed a trend toward improved hemoglobin A_1c_ levels in digital intervention groups compared to controls (SMD −0.96, 95% CI −2.03 to 0.11) but did not reach statistical significance (*I*²=97%). Meta-analysis for cognitive disorder DTx showed significant improvement in global cognitive function in DTx-treated participants (SMD 0.65, 95% CI 0.37-0.94), despite notable heterogeneity (*I*²=71.7%). The commercial landscape analysis identified 97 active DTx solutions, primarily targeting cognitive impairment (38 companies), ophthalmic diseases (30 companies), and respiratory diseases (5 companies). The regulatory review highlighted China’s reliance on general medical device policies under the National Medical Products Administration rather than DTx-specific regulations, with emerging regional innovation policies supporting industry growth. Pricing analysis revealed diverse reimbursement models, including value-based pricing, private insurance partnerships, and government-facilitated programs.

**Conclusions:**

China has made substantial progress in DTx development but still faces challenges in clinical trial standardization, regulatory approval, and reimbursement. Key factors driving DTx adoption include targeted indications, standardized clinical trials, streamlined regulation, and diversified pricing models. China’s experience provides valuable lessons for other countries with emerging digital health ecosystems as they develop DTx research, regulations, and integration strategies.

**Trial Registration:**

PROSPERO CRD42024615584; https://www.crd.york.ac.uk/PROSPERO/view/CRD42024615584

## Introduction

Digital therapeutics (DTx) encompass evidence-based interventions delivered via software or digital platforms to prevent, manage, or treat medical conditions, particularly major chronic diseases [1]. In China, DTx are defined as “evidence-based therapeutic interventions driven by high-quality software programs to treat, manage, or prevent a disease or disorder” [2]. These programs interact with patients through information (eg, text, images, and videos) and physical factors (eg, sound, light, electricity, and magnetic fields) [3]. Unlike traditional pharmaceuticals, DTx leverage digital tools like mobile apps, wearables, and virtual reality to enhance health outcomes [4]. These interventions are patient-facing software applications, which target behavioral change, cognitive training, or symptom management [5]. They thus far were primarily developed for indications such as chronic and neuropsychiatric diseases. DTx are gaining prominence in global health care systems [3] and can complement or even replace conventional medical approaches beyond basic health monitoring [6]. Since they are software-driven, DTx potentially allow more precise monitoring and measurement of patient progress and treatment adherence compared to pharmaceuticals [7]. For instance, DTx tailor treatments to individual needs, considering patient history, preferences, and real-time data from wearables or apps [8]. By modifying behaviors (eg, medication adherence and exercise routines) through digital apps, reminders, and feedback loops, DTx effectively manage chronic conditions by monitoring symptoms, providing health information, and promoting self-care [1]. They also enable clinicians to collect real-time patient data remotely, streamlining subsequent office visits [9]. Ultimately, DTx have the potential to reduce health care costs by personalized interventions, enhancing treatment engagement, and improving overall health outcomes, thus promising to transform health care delivery.

China’s rapidly aging population and escalating chronic disease burden (eg, cardiovascular diseases, diabetes, hypertension, and mental health disorders) necessitate early intervention [10-14]. The health care system faces challenges in managing this growing demographic, creating an urgent need for innovative solutions. DTx offer a promising approach, and China’s rapid growth in this area provides valuable lessons and examples for the rest of the world. With the increasing health care awareness, the DTx market in China has witnessed significant growth and interest. China’s DTx have grown exponentially, outpacing most Western countries, especially in health care areas directly linked to e-commerce [15]. The China DTx market was worth around US $0.5 billion in 2023 and is projected to reach US $2.92 billion by 2030, with a compound annual growth rate of 28.7% [16]. The evidence-based DTx market in China is worth around US $200-250 million, including hospital-facilitated, business-to-business products; pharmaceutical or medical technology collaboration products; and non–prescription-based, business-to-consumer products [15]. The Chinese government recognizes digital health as a key growth frontier and has actively supported its development through policies that facilitate clinical implementation, regulation, and commercialization. These efforts have attracted numerous enterprises, fostering the emergence of preliminary DTx industry clusters.

**Figure 1:**
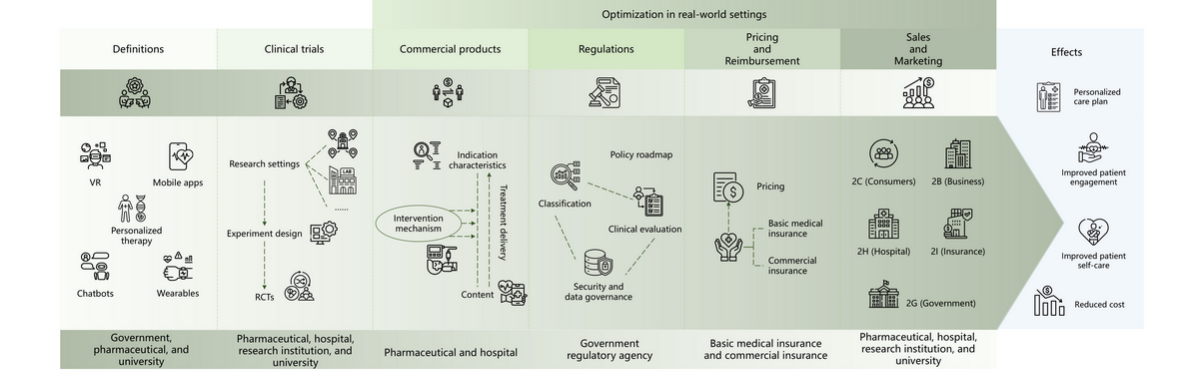
An overview of the digital therapeutic ecosystem in China. RCT: randomized controlled trial; VR: virtual reality.

While comprehensive reviews of the DTx ecosystem exist in regions like the United States, Europe, and South Korea [7], a holistic analysis of China’s DTx landscape remains absent. This represents a significant research gap, as understanding China’s approach to DTx requires examining the interconnections between clinical evidence, commercialization strategies, and regulatory frameworks. Previous studies have typically focused on isolated aspects of DTx development, failing to capture the complex interplay between these critical dimensions. Given the sector’s reliance on patient engagement, public trust, and regulatory navigation, an integrated assessment that examines these 3 dimensions together is essential not only for advancing China’s DTx ecosystem but also for providing valuable insights that could inform global DTx development (Figure 1).

To address these gaps, our study aims to conduct a comprehensive assessment of China’s DTx landscape through the integrated analysis of clinical trials, commercial products, regulatory frameworks, and reimbursement models. This multidimensional approach allows us to identify the key barriers and potential facilitators for DTx adoption in China, providing actionable recommendations to inform DTx research, development, adoption, and implementation in other countries. We document the trends and characteristics in key DTx domains in China, including intended indications, commercial products, clinical trials, regulatory frameworks, and reimbursement policies. Furthermore, we delved into the intricate interplay among research, development, clinical trials, and regulatory aspects, considering both established and emerging DTx solutions. Our analysis leads us to propose essential considerations for each DTx domain and advocate open dialogue to effectively position DTx as a standard medical practice worldwide.

## Methods

### Methodological Framework

Our study used a multimethod approach to comprehensively evaluate China’s DTx ecosystem. This integrated framework was necessary to capture the full spectrum of clinical evidence to market dynamics and policy environments. We combined systematic review and meta-analysis of clinical trials to assess treatment effectiveness, scoping review of commercial products to understand market trends, regulatory analysis through policy document review to examine governance frameworks, and pricing or reimbursement analysis to evaluate economic sustainability. This methodological integration enabled us to examine how these different domains interact and influence each other, providing a more holistic understanding than any single methodological approach could offer.

### Clinical Trials

To evaluate the advancement of DTx research and clinical trials in China, we conducted a systematic review and meta-analysis of relevant literature following the PRISMA (Preferred Reporting Items for Systematic Reviews and Meta-Analyses) 2020 guidelines [17]. Literature research was performed across 5 databases (PubMed, Google Scholar, IEEE, Web of Science, and ScienceDirect) from their respective inception dates until July 2024. The search was limited to English-language publications. The search strategy included terms such as “Digital therapeutics OR DTx,” “China,” “Digital health OR Healthcare,” and “Smartphone OR Application.” The detailed search strings for each database are provided in Tables S1 and S2 in Multimedia Appendix 1 [18-38].

Clinical trial studies were included if they met the following criteria: (1) DTx clinical trials conducted in China, (2) intervention-based studies, and (3) used DTx-related technologies. Studies were excluded if they were (1) nonresearch papers such as conference abstracts, editorials, or opinion papers; (2) case reports; and (3) observational studies. Two researchers (CH and XY) independently screened the titles and abstracts of the retrieved papers, and full-text papers were reviewed for eligibility. Disagreements were resolved through discussion with a third researcher (NJ).

For data extraction, a standardized form was developed to systematically collect information from each included study. The extracted data included publication year, indication, geographic location (province), type of study design, and type of clinical research study. This structured approach enabled a comprehensive analysis of trends and patterns in DTx clinical research across China. Details regarding meta-analysis of clinical trials, such as search strategies, selection criteria, and risk of bias, are present in Figure S1 and Tables S3 and S4 in Multimedia Appendix 1. The meta-analysis studies of clinical trials were registered at PROSPERO CRD42024615584 for cognitive disorder outcomes and CRD42024611857 for diabetes outcomes. This separation was necessary due to the distinct outcome measures and analytical approaches required for each condition. Meta-analyses were conducted using random-effects models due to expected heterogeneity between studies. The analyses were performed using R (version 4.3.2; R Foundation for Statistical Computing) with the *meta* package. Standardized mean differences (SMD) with 95% CIs were calculated.

### Commercial Products

To understand the development trends and characteristics of commercial DTx products in China, we conducted a scoping review and searched for commercial products mentioned in the National Medical Products Administration (NMPA) product library without any specific time restrictions in order to capture all relevant products until July 2024 [39]. We limited the category to domestic medical devices (registration) and used the following search terms: “software,” “digital therapeutics,” and “system.” Products were excluded based on their scope of application or intended use.

For the data extraction process, we systematically collected information on each eligible product, including the release year, indication, province, type of clinical research study, and regulatory classification. The same 2 researchers independently reviewed product documentation to ensure the accuracy and comprehensiveness of the extracted data. This systematic approach allowed us to identify patterns in product development, geographic distribution of DTx companies, and evolving trends in the commercial landscape. The synthesis of this data provided insights into the maturation of the DTx market in China and how it compares with global developments.

### Regulations

DTx regulations were examined in China to understand their impact on clinical trials, product development, and market adoption. We conducted a systematic document analysis using a thematic framework approach. The analysis covered 3 key regulatory domains: premarket approval pathways, technical review guidelines, and postmarket surveillance requirements. We browsed websites of relevant government institutions, including the State Council of the People’s Republic of China, the National Development and Reform Commission, the Ministry of Science and Technology of the People’s Republic of China, the NMPA, and the State Administration for Market Regulation. The inclusion criterion was whether the regulations were relevant to DTx and effective until July 2024. The same pair of 2 researchers independently analyzed the texts using a structured coding framework to identify policy hierarchies, regulatory requirements, and implementation timelines. The coded information was then synthesized into thematic maps illustrating the national and regional policy framework and the classification-based regulatory pathways for DTx products. This methodical approach allowed us to comprehensively document China’s evolving DTx regulatory ecosystem, including both national directives and regional innovation policies.

### Pricing and Reimbursement

To analyze DTx pricing and reimbursement models in China, we conducted a comprehensive review of public and private insurance policies, government initiatives, and commercial pathways. We examined official documents from health care authorities, insurance companies, and regional pilot programs. For regulatory documents, rather than using electronic database searches, we conducted a comprehensive manual review of all available policy documents on official government websites from January 1, 2025 to July 31, 2024. The review focused on 4 key aspects: pricing mechanisms, public health insurance coverage, private insurance integration, and alternative financing models. Information was collected from government websites, insurance company reports, and publicly available policy documents up to July 2024.

## Results

### Clinical Trials of DTx in China

**Figure 2:**
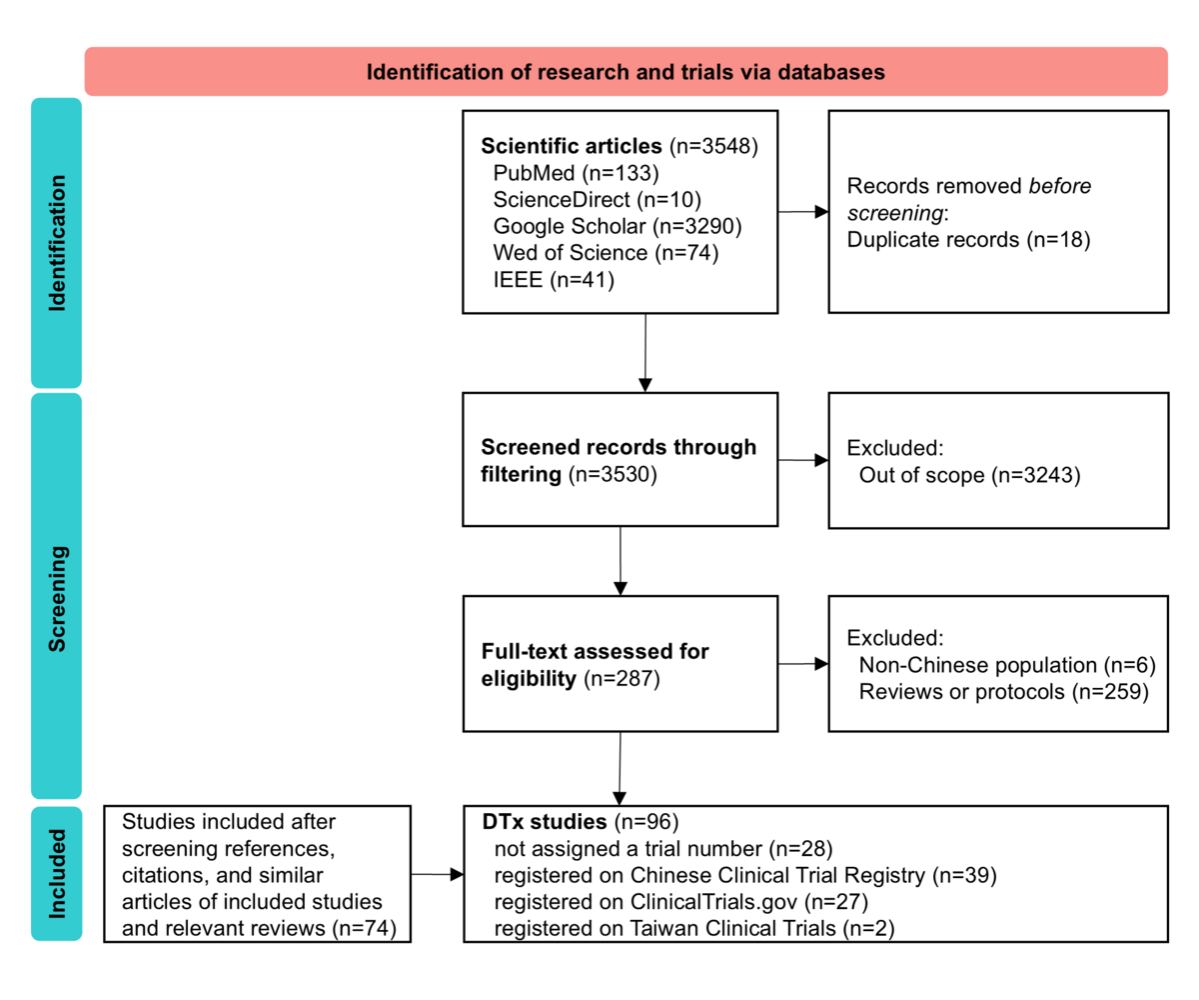
PRISMA flowchart for the search process related to clinical research and trials of digital therapeutics (DTx).

A total of 3548 literature records related to DTx in China were identified. After screening the full text based on the inclusion criteria, 96 clinical studies were included in the review (see Figure 2 for the PRISMA flow diagram). Of the 96 included studies, 68 (71%) were registered: 27 on ClinicalTrials.gov and 41 on the Chinese Clinical Trial Registry website. The remaining studies were included based on published papers. These studies were included to ensure a comprehensive analysis of all relevant clinical research on DTx in China, regardless of their registration status.

**Figure 3:**
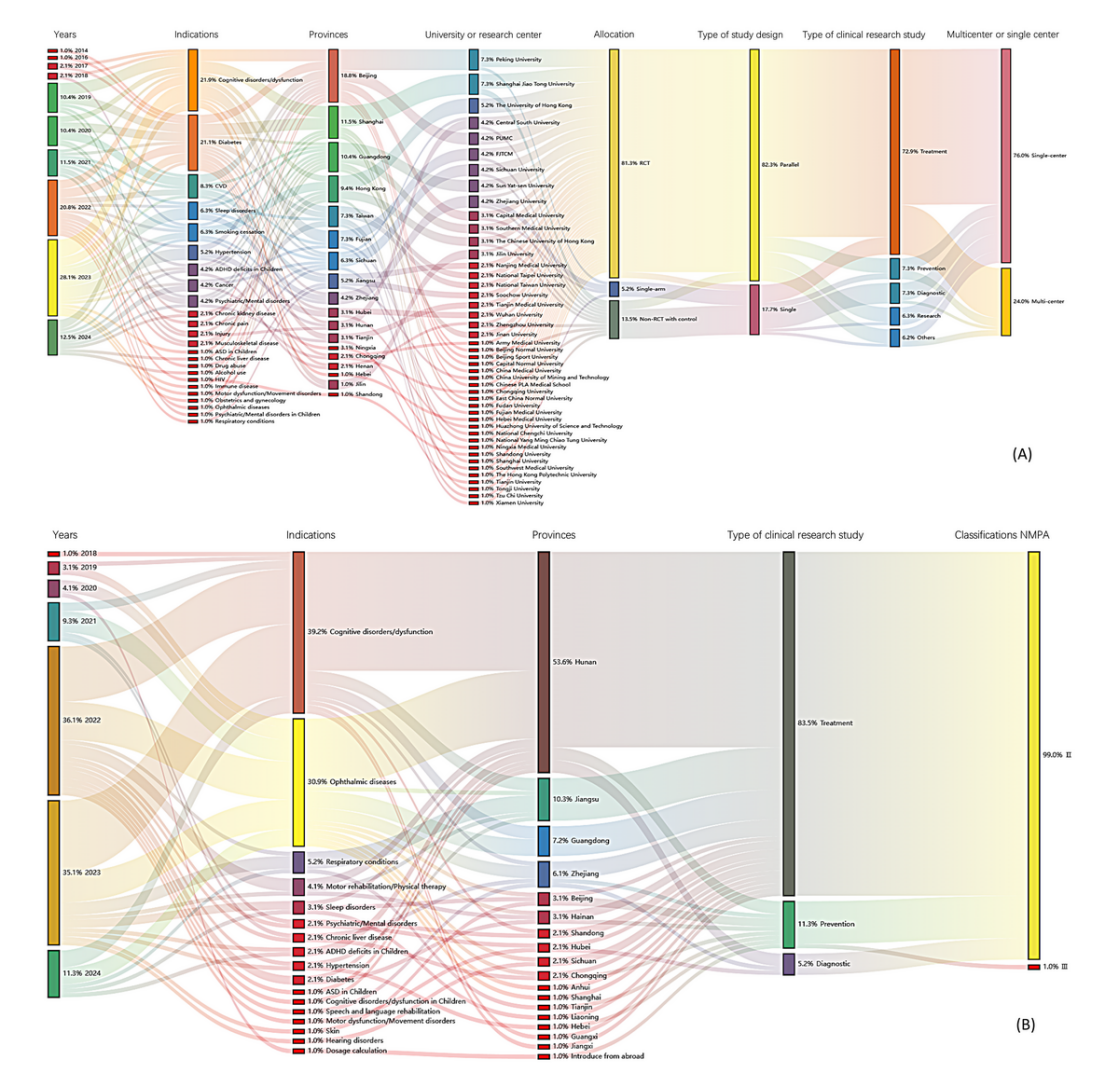
Sankey diagrams. (A) A Sankey diagram was used to analyze trends in indication, institution, province, trial type, intervention, and major outcomes for clinical trials. Between 2014 and 2024, a total of 96 clinical trials related to digital therapeutics (DTx) were identified. The clinical trials were conducted by a variety of institutions, including pharmaceutical companies, hospitals, research institutes, medical consortia, universities, and corporations. These trials were carried out in China, using interventions such as parallel, single, crossover, and factorial designs, with primary objectives that included treatment, research, prevention, and diagnosis. (B) A Sankey diagram was used to analyze trends in the types of indications, registered provinces, primary end points, and class types for commercial digital therapeutic devices (n=97). The investigation focused on products listed on the NMPA websites. Commercial DTx are primarily launched for indications related to neurology, psychiatry, ophthalmic diseases, chronic diseases, and respiratory conditions, with additional products available for hearing disorders, skin conditions, and sleep disorders. The primary objectives include treatment, prevention, and diagnosis. FJTCM: Fujian University of Traditional Chinese Medicine; NMPA: National Medical Products Administration; PUMC: Peking Union Medical College.

Figure 3A shows a Sankey diagram analyzing clinical trials presented through published research papers. This diagram shows the relationship between publication year, indication, province, type of study design, and type of clinical research study. It indicates that the types of indications have been diversifying over the years, illustrating that companies are prioritizing clinical trials more proactively. In addition, most clinical trials were conducted using randomized controlled trials (RCTs). For study design, the parallel design had the highest proportion. For the primary end point, treatment was the most common outcome, followed by diagnosis.

Of 96 clinical trials, 21 (22%) were related to cognitive disorders, followed by diabetes (20 trials, 21%), cardiovascular diseases (8 trials, 8%), sleeping disorders (6 trials, 6%), and smoking cessation (6 trials, 6%). Neuropsychiatric and chronic diseases account for the majority of indications. However, it has been shown that the scope of DTx is expanding with indications for diseases as follows: neurodegenerative diseases such as mild cognitive impairment and Parkinson disease; cancer diseases such as gynecologic cancer-related pain management; chronic diseases such as chronic kidney disease, liver disease, respiratory conditions, and hypertension; and neurological diseases such as autism spectrum disorders, depression, and anxiety; and sleep disorders.

For geographic distribution, Beijing conducted the most with 18 (19%) publications, followed by Shanghai with 10 (11.5%), Guangdong with 7 (10.4%), Hong Kong Special Administrative Region with 7 (9.4%), Taiwan with 7 (7.3%), Fujian with 6 (7.3%), Sichuan with 6 (6.3%), and Jiangsu with 4 (5.2%). Clinical research was conducted by various institutions, all in collaboration with universities and affiliated hospitals.

Risk of bias assessment using the Cochrane Risk of Bias 2 tool for RCTs revealed varying quality across the included studies. For cognitive disorders or dysfunction studies (Figure S1A in Multimedia Appendix 1), 3 studies demonstrated an overall low risk of bias [18-20], while 5 studies showed some concerns [21-25], and 2 studies presented a high risk of bias. Relative frequent sources of bias were in the randomization process (D1) and deviations from intended interventions (D2). For diabetes studies (Figure S1B in Multimedia Appendix 1), 5 studies demonstrated an overall low risk of bias [26-30], while 6 studies showed some concerns or high risk of bias [31-36]. Across both indications, measurement of outcomes (D4) and selection of reported results (D5) showed better methodological quality. These findings highlight the need for more rigorous trial design standards specific to digital interventions, particularly regarding randomization procedures and intervention implementation.

**Figure 4:**
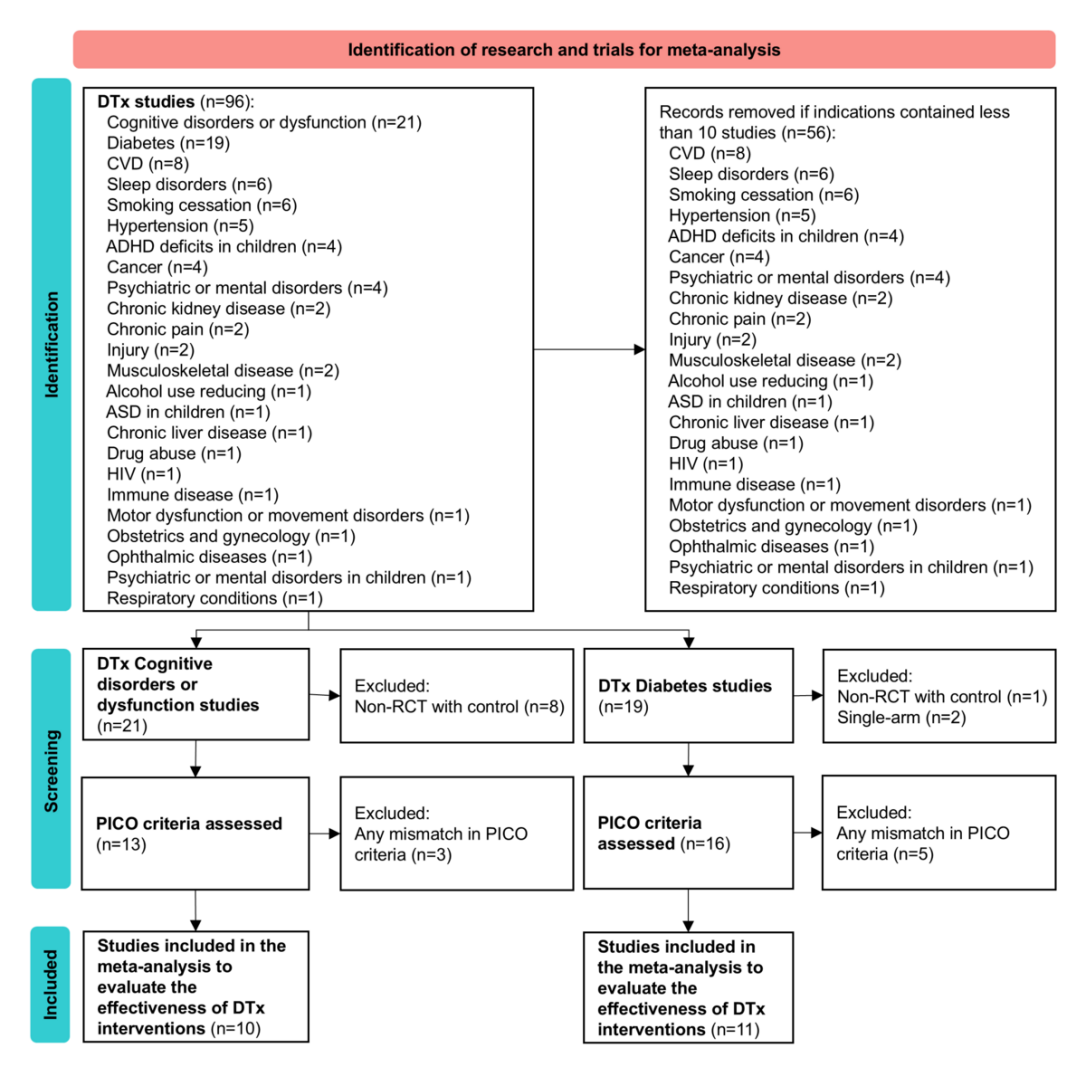
PRISMA flowchart for the selection process for two meta analyses of digital therapeutics (DTx) intervention. For meta-analysis of diabetes studies, studies were selected according to the following criteria: Population (P): adults diagnosed with type 2 diabetes mellitus, particularly those with poor glycemic control (HbA_1c_ ≥7%), regardless of disease duration; Intervention (I): digital therapeutic interventions, including by not limited to smartphone-based apps, WeChat-based interventions, and web-based telemedicine systems; Comparison (C): standard care or usual care, encompassing regular face-to-face clinical visits, traditional diabetes education, routine blood glucose monitoring, and standard lifestyle advice; and Outcomes (O): change in HbA_1c_ levels (%). Only full-text, peer-reviewed RCTs published in English were considered for inclusion. For meta-analysis of cognitive disorders and dysfunction studies, studies were selected according to the following criteria: Population (P): adults with a confirmed diagnosis of mild cognitive impairment or vascular cognitive impairment by a medical physician or based on accepted classification or criteria, excluding those with psychiatric issues, significant medical comorbidities, or conditions affecting cognitive test performance; Intervention (I): any digital health intervention, including but not limited to smartphone-derived interventions, PC-delivered interventions, tablet-delivered interventions, digital assistants, media players, and video game consoles; Comparison (C): active control groups receiving conventional education or usual care; and Outcomes (O): change in global cognitive ability measured via standardized neuropsychological tests such as MMSE and MoCA. Only full-text, peer-reviewed RCTs published in English were considered for inclusion. ADHD: attention-deficit/hyperactivity disorder; ASD: autism spectrum disorder; CVD: cardiovascular disease; HbA_1c_: hemoglobin A_1c_; MMSE: Mini-Mental State Examination; MoCA: Montreal Cognitive Assessment; PICO: Population, Intervention, Comparison, and Outcomes; RCT: randomized controlled trial.

**Figure 5:**
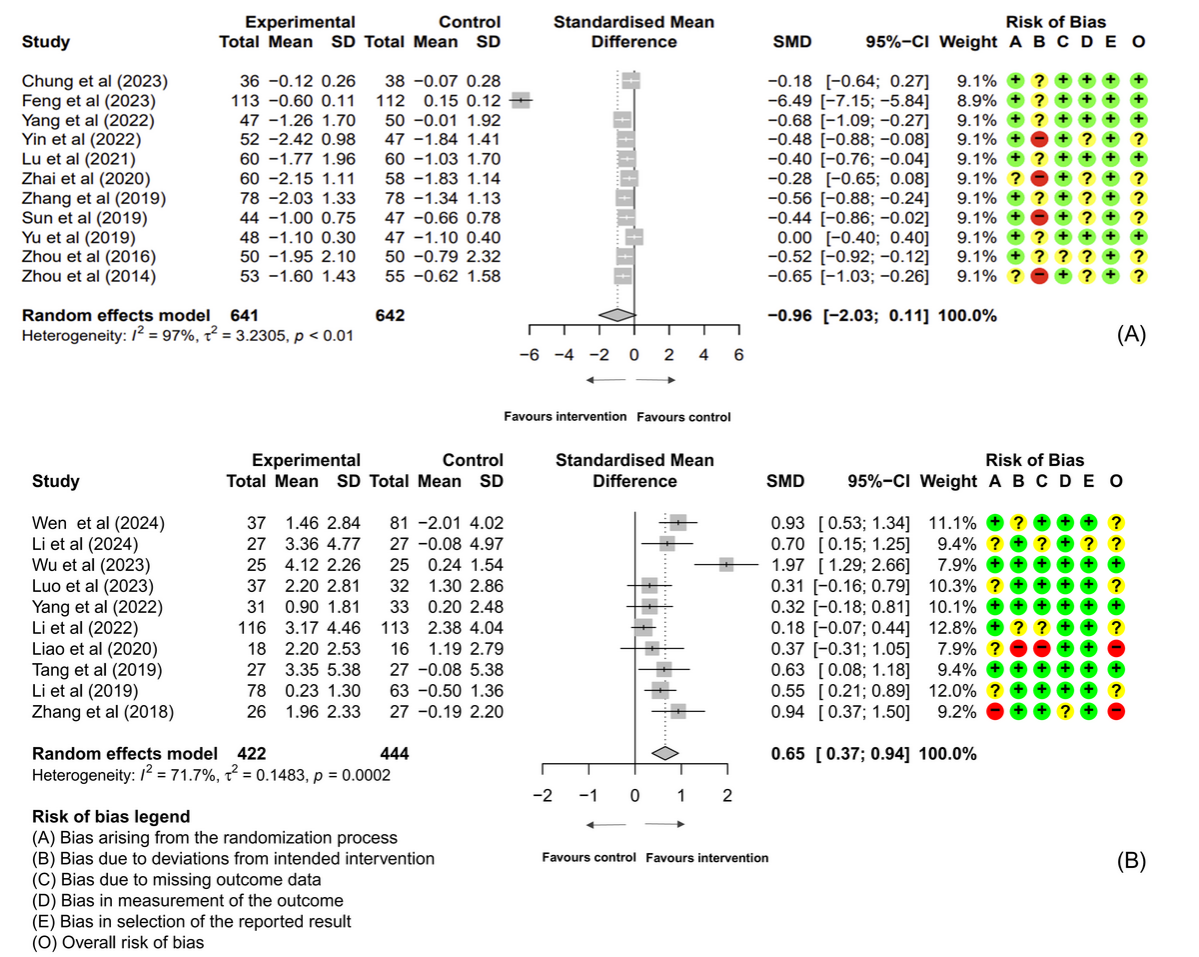
Meta-analysis for digital therapeutics (DTx) interventions for diabetes and global cognitive function. (A) A difference in HbA_1c_ (expressed as a percentage) between the DTx intervention and the usual care group in Chinese patients with T2DM. The meta-analysis showed a trend toward improvement in HbA_1c_ levels among participants in the DTx intervention group compared to the control group, approaching but not reaching statistical significance. The data from 11 RCTs (involving 1283 participants) present substantial heterogeneity. (B) Forest plot of mean difference in global cognitive function (measured by Montreal Cognitive Assessment and Mini-Mental State Examination) between the DTx intervention and the usual care group in Chinese patients with cognitive impairment. A statistically significant greater increase in global cognitive function scores is seen in the DTx intervention group compared with the control group. The data from 10 RCTs (involving 866 participants) present substantial heterogeneity. The size of the squares indicates the weight of the evidence from each of the studies. Studies with CI (horizontal line) crossing 0 (vertical line) are inconclusive. Powerful studies (those with more participants) have narrower CIs. The diamonds represent the summary effect sizes in the overall sample, with the width of the diamond indicating the 95% CI. HbA_1c_: hemoglobin A_1c_; RCT: randomized controlled trial; SMD: standard mean difference; T2DM: type 2 diabetes mellitus.

Due to the heterogeneity of study outcomes, we only chose DTx for diabetes and cognitive disorders for meta-analyses. The PRISMA flowchart for the selection process is shown in Figure 4. For diabetes, 11 studies (1283 participants) were included in the meta-analysis on hemoglobin A_1c_ (HbA_1c_). Of the included studies in the meta-analysis, 3 DTx interventions were assessed as high risk of bias, and 5 as low. The meta-analysis showed a trend toward improvement in HbA_1c_ levels among participants in the DTx intervention group compared to the control group (SMD −0.96, 95% CI −2.03 to 0.11; Figure 5A [18-38]), approaching but not reaching statistical significance. This effect size suggests potential clinical relevance, as a reduction of approximately 1% in HbA_1c_ is generally considered meaningful for diabetes management. However, substantial heterogeneity was observed across studies (τ^2^_10_=3.23; *P*<.001; *I*²=97%). For cognitive disorders, 10 studies (866 participants) were included in the meta-analysis on global cognitive function measured by the Montreal Cognitive Assessment and Mini-Mental State Examination. This meta-analysis showed a significant improvement in global cognitive function among participants in the DTx intervention group compared to the control group (SMD 0.65, 95% CI 0.37-0.94; Figure 5B). The heterogeneity between the studies was statistically significant and considerable in magnitude (τ^2^_9_=0.15; *P*<.001; *I*²=71.7%). Three studies were rated as low risk of bias, and 2 studies may face a high risk of bias.

### Commercialization of DTx

**Figure 6:**
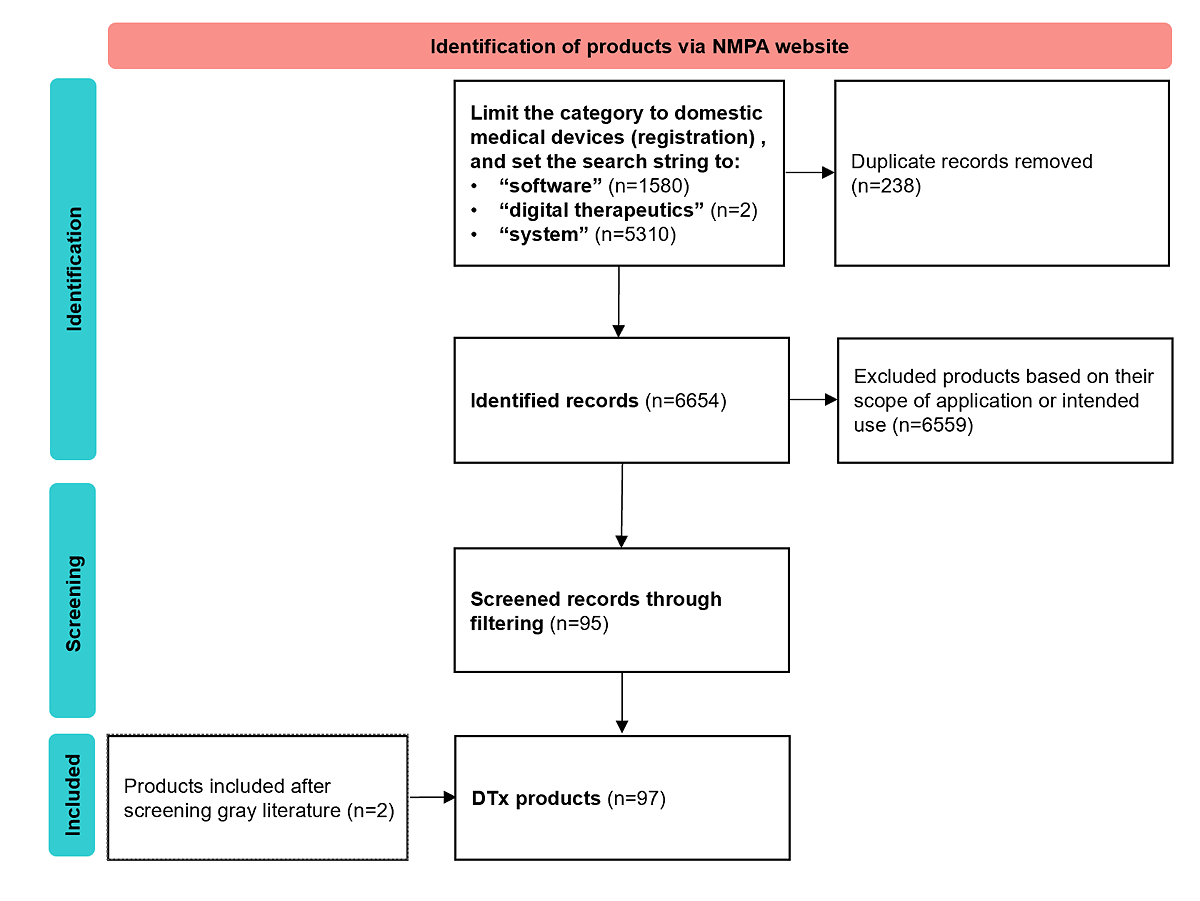
PRISMA flowchart for the search process related to digital therapeutics (DTx) products in China. NMPA: National Medical Products Administration.

A systematic search of DTx-related commercial products in China revealed that DTx initially focused on a limited range of conditions reliant on behavioral interventions, such as addiction and mental health. Recent DTx launches cover diverse indications, including diabetes, hypertension, respiratory diseases, hepatic conditions, sleep disorders, and attention-deficit/hyperactivity disorder. Cognitive impairment was the most active field, with 38 companies involved, followed by ophthalmic diseases (30 companies) and respiratory diseases (5 companies). Together, these 3 fields involved over 73 companies, totaling 97 and accounting for more than half of the DTx market (Figure 6).

A representative example of commercialized DTx is Six Brain (BrainAu), which is the first NMPA-cleared DTx for computerized cognitive training in older adults. Six Brain offers prescription behavioral medicine, providing multidomain, adaptive cognitive training to enhance global cognitive function, executive function, and brain functional connectivity [18]. Another NMPA-cleared DTx is BBRT (BestCovered). BBRT combines cognitive behavioral therapy with a multidomain intervention, including diet, exercise, cognitive training, vascular risk monitoring, and professional web-based counseling services. It aims to prevent cognitive decline in at-risk older individuals. Additionally, the Shukang app serves as a DTx for cardiac rehabilitation. The app offers domiciliary, mobile app–guided, and telemonitored programs to improve physical fitness, adherence, and health beliefs among patients who have undergone ablation for atrial fibrillation [40]. DTx differ from traditional medications as they deliver treatment through applications on computers or smartphones. While the underlying technologies are similar across indications, treatment outcomes depend on content and application methods (Table 1). Companies customize content for specific indications on their platforms, collaborating with research institutions like universities and hospitals to expand DTx to various conditions through existing or new partnerships.

Figure 3B shows a Sankey diagram of commercialized DTx products in China with release year, indication, province, type of clinical research study, and regulatory classification. The number of commercial products released has an increasing trend from 2018 to 2023. A total of 97 products received NMPA clearance from 2018 to the first half of 2024. Many of these products initially released were related to neurological diseases, ophthalmic diseases, psychiatric diseases, and endocrine diseases. Products released after 2022 were observed to be related to respiratory diseases, probably due to COVID-19. Most DTx emphasize treatment and rehabilitation, highlighting the benefits of DTx in enhancing adherence and improving patient experience. Among the approved DTx products in China, the majority are registered as class II medical devices. There is only 1 DTx product classified as class III, designed for medication dosage calculation.

**Table 1:** A comprehensive overview of primary intervention mechanisms of commercialized DTx^a^.

Diseases	Indications	Intervention mechanism of DTx	Mode of delivery	Unique DTx benefits
Psychiatric or mental disorders	ADHD^b^, depression, insomnia, addiction, anorexia nervosa, and substance abuse	Cognitive behavioral therapy and behavioral activation	Smartphone apps, web-based platforms, VR^c^ therapy, and wearable devices	Boosting therapy effectiveness and patient outcomes with software-hardware integration.
Neurological diseases	AD^d^, PD^e^, multiple sclerosis, epilepsy, and migraine	Cognitive behavioral therapy, biofeedback therapy, and relaxation therapy	Smartphone apps, web-based platforms, VR therapy, wearable motion sensors, and biofeedback devices	Boosting therapy effectiveness and patient outcomes with software-hardware integration.
Endocrine and metabolic diseases	Diabetes and metabolic syndrome	Exercise and nutrition therapy	Smartphone apps, wearable devices, connected glucose monitors, AI^f^-driven analytics, web-based platforms, and telemedicine integration	Enhancing patient self-management with long-term health tracking
Cancer	Cancer complications	Pharmacokinetic precision medication and disease course management	Smartphone apps, web-based platforms, remote patient monitoring, AI-driven analytics	Leveraging AI and big data to analyze in vitro drug data, personalizing treatments by understanding individual drug responses and optimizing patient outcomes
Respiratory diseases	Chronic obstructive pulmonary disease, and asthma	Pharmacokinetic precision medication monitoring	Smartphone apps, wearable devices, web-based platforms, inhaler sensors, and VR-based rehabilitation	Leveraging AI and big data to analyze in vitro drug data, personalizing treatments by understanding individual drug responses and optimizing patient outcomes
Cardiovascular diseases	Heart failure and hypertension	Pharmacokinetic precision medication monitoring and disease course management	Smartphone apps, wearable devices, remote monitoring platforms, connected BP^g^ cuffs, and telemedicine integration	Leveraging AI and big data to analyze in vitro drug data, personalizing treatments by understanding individual drug responses and optimizing patient outcomes
Ophthalmic diseases	Myopia, amblyopia, and strabismus	Interfering with synapses in the central neural circuit of vision	VR headsets, eye-tracking screen devices, and specialized digital visual simulation tools	Boosting brain plasticity and visual performance with focused, noninvasive stimulation of the visual cortex, resulting in enhanced binocular vision
Musculoskeletal diseases	Musculoskeletal pain and osteoarthritis	Exercise therapy	Smartphone apps, wearable motion sensors, camera-based tracking, VR-based rehabilitation, and digital physical therapy platform	Enhancing the therapeutic experience for both providers and recipients

^a^DTx: digital therapeutics.

^b^ADHD: attention-deficit/hyperactivity disorder.

^c^VR: virtual reality.

^d^AD: Alzheimer disease.

^e^PD: Parkinson disease.

^f^AI: artificial intelligence.

^g^BP: blood pressure.

### Regulation of DTx

**Figure 7:**
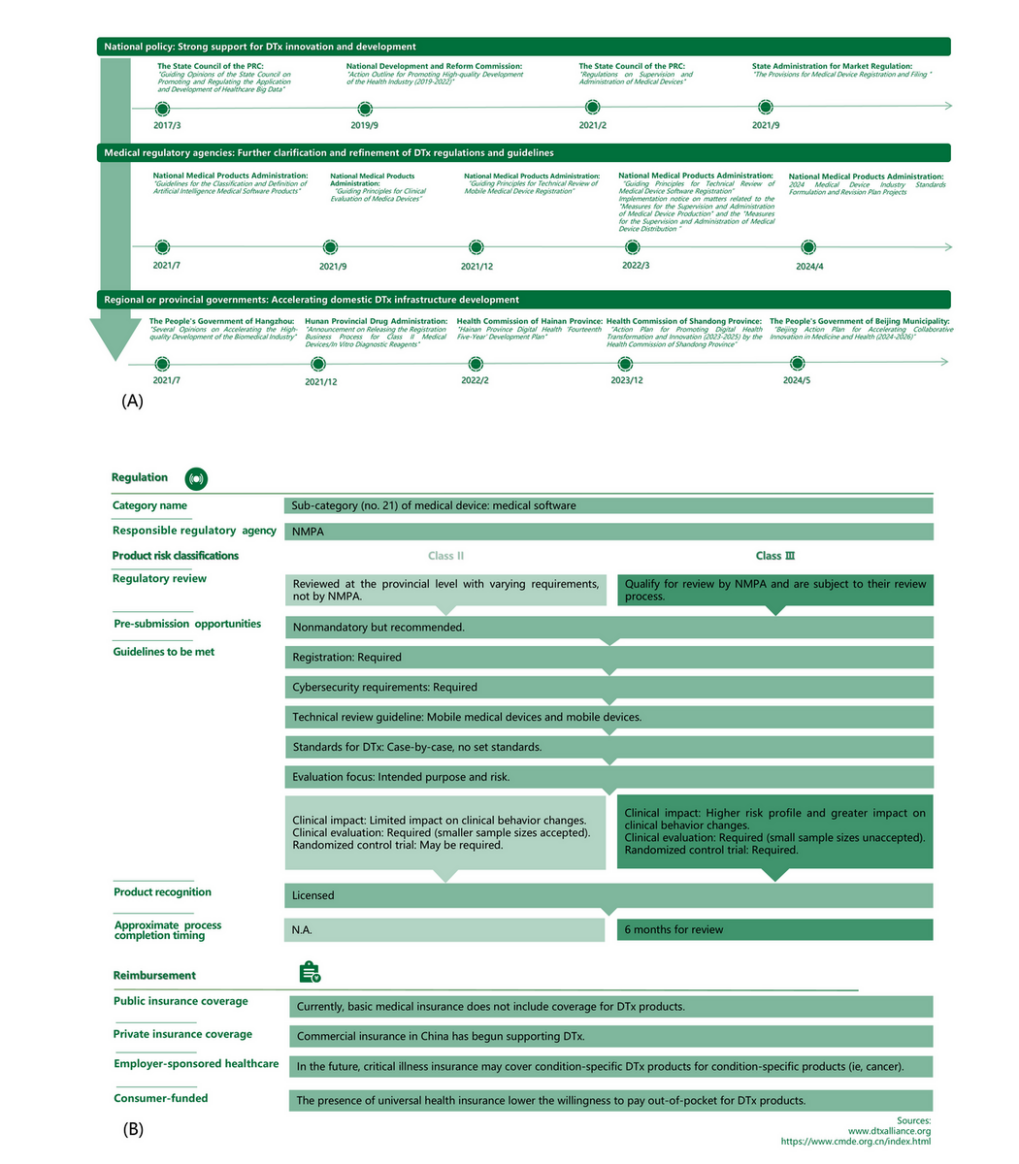
DTx policy development in China: (A) DTx innovation development policy map and (B) regulation and reimbursement framework of DTx in China. DTx: digital therapeutics; NA: not applicable; NMPA: National Medical Products Administration; PRC: People’s Republic of China.

At present, there is no specific regulation or issued guidance principles for DTx products in China. The regulation and registration of similar products follow the relevant policies and procedures for medical device software approval (Figure 7A). The regulation and registration policy framework for DTx is guided by national policies [41-44] and specific procedures set forth by the NMPA [45-50]. These regulations cover various aspects of DTx development and deployment, including registration, classification, clinical evaluation, production, distribution, and software review. Additionally, to foster innovation and growth in the DTx industry, several provinces have implemented their own innovative policy frameworks and incentives, demonstrating a varied regulatory landscape across the country.

### Classification

In China, DTx products must be registered with the NMPA or local counterparts based on their classification (Figure 7B). Most DTx products are classified as class II medical devices, which offer more development flexibility but are subject to stricter regulations than class I devices, though less stringent than class III [50]. The DTx products are generally classified as software as medical device under existing categories, although these may not fully capture the specifics of DTx. General software regulations, encompassing technical review and classification, are applicable; however, the guidelines specific to DTx remain comparatively lenient.

When compared to the United States, both China and the United States adopted a risk-based classification system for DTx, categorizing products into classes I, II, and III based on the intended use and risk level. In the United States, DTx is regulated by the Food and Drug Administration’s Center for Devices and Radiological Health under the Mobile Medical Applications framework. Class I devices are subject to general controls; class II devices typically require 510(k) clearance; and class III devices, which pose the highest risk, undergo premarket approval [51]. In contrast, China’s NMPA follows a similar tiered classification approach, but regulatory pathways and enforcement mechanisms differ. Key differences include regulatory oversight, where China’s approach remains more prescriptive, approval pathways, which rely on preexisting classification rules rather than de novo pathways, and postmarket surveillance, where China enforces stricter compliance measures to ensure long-term safety and efficacy.

### Clinical Evaluation

According to the “Measures for the Registration and Filing Management of Medical Devices” (State Administration for Market Regulation decree no. 47) [44], clinical evaluation is typically required for medical device registration to confirm safety and effectiveness unless specific exemptions apply. For DTx products, the “Catalogue of Medical Devices Exempt from Clinical Trials” (notice no. 71 of the NMPA in 2021) [52] allows exemptions for certain class II software, such as trace analysis and clinical management software. However, determining whether a product qualifies for exemption should involve a thorough algorithmic assessment and expert consultation. Clinical evaluation can be conducted through either clinical trials or by analyzing existing literature and data from similar devices.

### Security and Data Governance

DTx in China handle extensive personal data from medical services like disease prevention and treatment. Regulations require companies to disclose data processing rules, implement robust security measures, address vulnerabilities promptly, and conduct regular risk assessments with reports to authorities. In 2022, the NMPA updated the “Guiding Principles for Technical Review of Medical Device Network Security Registration” [53], requiring classes II and III medical devices with network connectivity to ensure security throughout their life cycle, from design to maintenance. These measures safeguard data, ensure device effectiveness, and support the growth of the DTx industry. In the same year, the NMPA Medical Device Technical Review Centre spearheaded the establishment of a “DTx Working Group” within the Artificial Intelligence (AI) Medical Device Innovation Cooperation Platform [54] to advance the exploration of AI regulatory frameworks in DTx.

### Pricing and Reimbursement of DTx

#### Pricing

In China, there is no well-defined framework for determining the prices of digital health apps. Additionally, there is no centralized committee responsible for setting national-level prices for these solutions. Instead, pricing negotiations occur at the hospital level, where each hospital collaborates with digital health manufacturers to reach agreements. Pricing mechanisms were found to be at different stages of development across China. For instance, Hainan deploys on a fee-for-service basis and determines the price of DTx in the context of the health care process in which they are deployed [55], and it is transiting to a value-based pricing system [55].

When comparing globally, reimbursement models differ significantly across countries (Table 2). In the United States, DTx reimbursement is primarily private insurer-driven, with some products covered under Medicare or Medicaid through current procedural terminology codes and value-based agreements [51]. Europe has structured models, such as Germany’s Digitale Gesundheitsanwendungen (DiGA), which provides temporary fast-track reimbursement, and France’s Patient Engagement and Communication Application Network system, which offers conditional reimbursement [56-58]. Canada and Australia rely on provincial or mixed public-private funding, with decentralized reimbursement decisions [59,60]. China’s reimbursement framework remains underdeveloped, with limited national coverage but growing private insurer involvement and pilot programs exploring new funding mechanisms. Despite these differences, similarities in risk-based classification and expanding payer coverage suggest the potential for global harmonization. International collaboration on DTx value assessment, health economic evaluations, and regulatory alignment could support broader adoption. Models like DiGA have influenced other regions, and a structured framework could accelerate DTx reimbursement in China. A standardized evaluation system would facilitate market integration, improve accessibility, enhance global DTx adoption, and drive innovation in digital health care solutions.

**Table 2:** DTx^a^ pricing model across different regions.

Region	Reimbursement model	Coverage approach	Challenges	Potential for harmonization
United States	Private insurers, Medicare or Medicaid coverage, and CPT^b^ codes for some DTx	Case-by-case evaluation by private insurers and emerging value-based pricing	Lack of standardization across payers and slow adoption of prescription DTx	Standardizing payer criteria and wider Medicare adoption
Europe	National frameworks in some countries (eg, Germany's DiGA^c^, France's PECAN^d^), others rely on regional or national health services	Temporary reimbursement while gathering evidence (Germany), conditional models in France	Varying national policies, not all countries have structured reimbursement	DiGA-style frameworks could expand to other EU countries
Canada	Provincial-level assessments, limited national coordination, employer-based insurance	Fragmented provincial funding, some employer and private coverage	No national framework, digital health products evaluated on a case-by-case basis	Adoption of a unified evaluation system across provinces
Australia	Mixed public-private model, some Medicare support for digital health, and private insurer involvement	Medicare covers some telehealth services; private insurers fund digital health	No centralized DTx policy, slow public adoption	National DTx reimbursement framework development
China	Limited national coverage, pilot reimbursement programs, growing role of private insurers	Primarily out-of-pocket or private insurance, limited government funding	Government insurance yet to cover DTx, regulatory pathways still evolving	Potential for structured reimbursement similar to Europe’s DiGA model

^a^DTx: digital therapeutics.

^b^CPT: Current Procedural Terminology.

^c^DiGA: Digitale Gesundheitsanwendungen.

^d^PECAN: Patient Engagement and Communication Application Network.

#### Eligibility and Mechanism of Reimbursement Through Public Health Insurance

China does not have a specific reimbursement process for DTx, nor established criteria for assessment. In digitally advanced areas, reimbursement decisions for DTx are driven locally by hospital groups. For example, Shanghai is integrating DTx into its comprehensive medical insurance as a tool for screening and disease management [61], while Hainan is encouraging the inclusion of DTx in medical service specifications and fee structures [62]. Two reimbursement archetypes could be derived: either reimburse the digital health solution itself or reimburse the clinical pathway that the digital health solution is a part of.

#### Financing DTx Through Private Health Insurance

We found an array of DTx solutions in China that are financed through private health insurance. This involves insurance providing payment and management for medical care, while medical services guide and interact with insurance. Constructing a closed loop for “preventive medicine” benefits both insurance and DTx by creating a positive incentive cycle. Insurance companies are increasingly using DTx to collect patient data and develop more targeted products. For example, ZhongAn Insurance launched a cognitive protection insurance product that includes BBRT therapy [63]; Miao Health introduced a product that incorporates DTx for hypertension management [6]; and Huzhibao offers comprehensive coverage for Alzheimer disease management, including eye movement screening, mild cognitive impairment blood biomarker testing, DTx intervention services, and medication reimbursement [61]. By integrating these services, DTx tools can provide comprehensive care for insurance beneficiaries, spanning from early detection and prevention to treatment, rehabilitation, and long-term management.

#### Financing DTx Outside Health Insurance

Examples of DTx financing independent of health insurance are prevalent in China, primarily focusing on public health and civil affairs. The dominant commercial pathway involves government-facilitated access for end users, following models such as business-to-government-to-consumer or business-to-hospital-to-consumer models. For instance, Amity Brain Health, in collaboration with tertiary hospitals in Fujian Province and Shenzhen City, has launched a pilot program for cognitive prevention and treatment, aiming to build a multilayered service system. This initiative aligns with government goals of improving health care accessibility and efficiency by addressing challenges such as limited screening, delayed diagnosis, and high treatment costs. Rapid screening, remote consultations, and home-based digital exercises provide comprehensive disease management, supporting the government’s efforts to enhance public health and reduce the burden of cognitive disorders [64].

## Discussion

### Principal Findings

Our study offers a comprehensive exploration of the DTx ecosystem in China. We summarize the following key lessons: (1) targeted indications and technology components; (2) standardized clinical trials; (3) streamlined regulation and approval process; and (4) diversified pricing and reimbursement models. These insights offer valuable lessons for other countries, particularly low- and middle-income nations that are looking to leverage DTx to improve health care outcomes and enhance population health.

#### Key Insight 1: Targeted Indications and Technology Components

Our analysis revealed strategic patterns in China’s DTx indication selection and technology deployment. Initially, DTx in China development primarily targeted chronic diseases like diabetes, cardiovascular conditions, and chronic pain; ophthalmic diseases such as myopia, amblyopia, strabismus, asthenopia, and postoperative rehabilitation; sleep disorders like insomnia; mental health challenges encompassing depression, anxiety, attention-deficit/hyperactivity disorder, and mild cognitive impairment; and substance abuse including smoking cessation. While these areas remain core to DTx applications, recent research indicates a growing trend toward more specialized DTx solutions tailored to specific disease subpopulations and clinical needs [1]. DTx is being used in a growing number of medical fields, including oncology, neurology, gynecology, urology, gastroenterology, orthopedics, pulmonology, immunology, otolaryngology, and infectious diseases. It also extends to various conditions like Parkinson disease, hepatitis B, and multiple sclerosis. A common trend of these emerging DTx domains in China is evident: DTx tends to be prioritized in areas where they can directly implement cognitive behavioral interventions or serve as complementary tools for medical professionals supporting doctors’ treatment and follow-up management. This aligns with the prediction that DTx would be preferentially adopted in fields with high demands and potential for cognitive behavioral therapeutic approaches [65]. Table S2 in Multimedia Appendix 1 presents a classification of DTx products based on their therapeutic indications, including both commercially available and investigational treatments.

The technology deployment for DTx in China shows distinctive characteristics shaped by the country’s digital infrastructure. The near-universal smartphone penetration and advanced mobile payment ecosystems have enabled a mobile-first DTx strategy, enabling real-time patient monitoring, personalized feedback, and improved adherence. This enhances cognitive-behavioral interventions and disease management, optimizing outcomes through remote monitoring and AI-powered decision support [51,66]. This approach has facilitated rapid scaling and accessibility of DTx solutions across diverse geographic regions and socioeconomic groups, offering valuable lessons for other large countries with significant urban-rural divides.

A fundamental aspect of DTx is content, which manifests in diverse forms such as virtual reality, gaming elements, web-based monitoring, and digital assessments. The therapeutic efficacy of a DTx is significantly influenced by the nature and design of its content. To optimize patient engagement and treatment outcomes, content development necessitates careful consideration of factors including target condition, patient demographics, and cultural context. Evolving societal values, cultural norms, and customs can also alter patient perceptions of digital content over time, thereby impacting the effectiveness of DTx. While this dynamic is difficult to standardize, it underscores the inherently changing nature of DTx efficacy. Clinician expertise has traditionally driven content creation; however, the integration of AI into treatment content is a burgeoning trend that warrants close attention [67,68]. Furthermore, there is a growing emphasis on the development of human-computer interaction and neurofeedback technologies to enhance the effectiveness [54]. China’s experience in this area demonstrates the critical importance of cultural adaptation in content development—a lesson particularly relevant for other countries with strong cultural traditions and diverse populations seeking to develop locally appropriate DTx solutions.

The efficacy of DTx is contingent upon users’ digital literacy and cognitive capabilities. Strong digital skills in using smartphones and computers are associated with enhanced content comprehension, engagement, and ultimately, therapeutic outcomes. However, the digital divide, particularly pronounced in China’s urban-rural divide, poses a significant challenge, potentially exacerbating existing health disparities. While direct evidence linking these factors to DTx outcomes in China is scarce, research on digital media suggests that age, education, and income impact user acceptance and engagement with digital content [69,70]. Given the parallels between DTx and other digital platforms, it is reasonable to infer that similar factors likely influence DTx’s effectiveness.

China’s experience in addressing this digital divide offers important lessons in designing inclusive DTx solutions. Equitable design principles that consider the specific needs of different regions have been essential to mitigate these disparities in the Chinese context. Patient engagement, influenced by sociocultural and demographic factors, is critical to DTx success [7], and China’s approaches to enhancing accessibility across diverse geographic regions and socioeconomic groups provide valuable insights. Countries with similar demographic and geographic diversity can learn from these strategies for tailoring interventions to diverse populations, maximizing the benefits of DTx while minimizing the risk of widening existing health inequities.

#### Key Insight 2: Standardized Clinical Trials

The predominant study design in our DTx research analysis was RCT, accounting for over 80% of included studies (78/96, 81%). This reflects China’s commitment to rigorous clinical validation of digital interventions, which sets an important precedent for other countries developing their DTx evaluation frameworks. However, challenges persist particularly in establishing suitable control groups for multicenter trials. Among the clinical research investigated in this study, only 21 multicenter clinical research studies were found. Moreover, the software nature of DTx complicates the creation of effective placebos, hindering the maintenance of blind conditions. Consequently, a tailored RCT framework is essential to address the unique characteristics of DTx. China’s experience highlights the need for adaptations to conventional clinical trial methodologies when evaluating digital interventions—a challenge faced globally. Due to the heterogeneity in outcomes, exposure, quantity, and quality of studies, meta-analysis was often infeasible or insignificant for many indications in our study. This underscores the field’s relative novelty and the urgent need for standardized methodologies and reporting guidelines.

While RCTs are essential for establishing treatment efficacy, real-world data are crucial for evaluating how interventions perform in practical settings. China’s approach to resolving these methodological challenges, including the emerging emphasis on real-world evidence to complement RCTs, offers valuable insights for other countries grappling with similar issues in DTx evaluation. Postmarket surveillance by both industry and academia is necessary to monitor the ongoing safety and effectiveness of approved DTx. The evolution of China’s clinical trial standards for DTx could serve as a reference point for countries developing their own evaluation frameworks, particularly those with limited experience in digital health assessment. By emphasizing evidence-based advantages, DTx may foster increased user acceptance and compliance in China. Subsequent to successful clinical trials, the industry should prioritize product dissemination, accelerate clinical integration, and expand community outreach. Thus, DTx may surmount adoption barriers and realize its full potential in improving patient outcomes and transforming health care delivery.

#### Key Insight 3: Streamlined Regulation and Approval Process

Although the NMPA has not yet issued clear guidelines specifically for DTx, approvals and registrations are increasing, and the potential for long-term growth is substantial. The regulatory landscape in China demonstrates a pragmatic approach that balances innovation with safety considerations. Unlike the United States (eg, De Novo, 501(k), and 501(k)exemption) [71] and the European Union (eg, DiGAs in Germany) [56,57], China lacks a dedicated DTx regulatory pathway. This approach differs notably from the more structured frameworks seen in these regions, offering an alternative model that may be more appropriate for countries with developing regulatory infrastructures.

Establishing an industry association to define DTx and distinguish them from other health management software is crucial for industry alignment. A unified conceptual framework can guide the creation of a comprehensive classification and definition system, clear approval standards, expedited reviews, and a streamlined approval process to assist companies in navigating product registration and entering the market more rapidly.

The regionalized innovation in regulatory approaches seen across different Chinese provinces provides a unique experiment in regulatory flexibility. To accelerate industry growth, local governments are increasingly competing to implement innovative incentives and create priority approval channels for DTx products [72]. This decentralized approach to regulation could be particularly instructive for other large countries with diverse regional health care needs and capabilities. With government leadership, local authorities can actively foster industry development, implement supportive policies, and create priority approval channels to expedite the registration and approval of DTx products.

China’s experience suggests that countries can make significant progress in DTx adoption even without fully developed DTx-specific regulatory pathways, provided there is sufficient flexibility within existing medical device regulation frameworks. This insight is especially valuable for countries that may lack the resources to develop comprehensive new regulatory frameworks specifically for digital health. Additionally, regulators in China carefully observe the DTx industry. If the use of new technologies such as AI increases, it will be necessary to develop standards for safety and efficacy verification [46,67,73].

#### Key Insight 4: Diversified Pricing and Reimbursement Models

China has pioneered innovative approaches to DTx reimbursement, with local governments taking the lead in shaping pricing and coverage strategies. These regional efforts reflect the complexity of navigating the fragmented reimbursement landscape while addressing challenges such as user acquisition, policy disparities, and cost-effectiveness evaluations. Local initiatives have leveraged direct-to-consumer sales, insurance-based coverage, and government funding to create tailored models that align with regional healthcare priorities [5].

The diversity of reimbursement models observed across different Chinese regions—from direct integration into public health insurance in Shanghai [61] to value-based approaches in Hainan [62]—offers a natural experiment in financing strategies that other countries can learn from. These varied approaches demonstrate that reimbursement pathways can be adapted to local health care financing systems and priorities, rather than requiring a one-size-fits-all approach. A value-based approach, focusing on clinical outcomes and cost-effectiveness, underpins these efforts. Health economic and outcomes research has been instrumental in quantifying the impact of DTx, such as reductions in hospitalizations and medication reliance, providing a solid basis for outcomes-based contracts and customized pricing strategies.

China’s experience with private insurance integration is particularly instructive for countries with mixed public-private health care systems. The creation of “closed loops” linking preventive medicine, treatment, and insurance reimbursement provides innovative models for sustainable DTx financing that extends beyond traditional fee-for-service approaches. By aligning incentives and fostering collaboration among government agencies, insurers, and health care providers, these local programs have driven significant innovation in reimbursement strategies [58].

The business-to-government-to-consumer and business-to-hospital-to-consumer models developed in China offer alternative commercialization pathways that could be especially relevant for countries where direct-to-consumer digital health markets are underdeveloped. Notably, local governments have introduced targeted policies, including pilot programs and dedicated reimbursement codes, to streamline DTx integration into their health care systems. This reimbursement for DTx is integrated into the remuneration of broader health care processes, aligning more effectively with diagnosis-related group payment models for health care.

These intermediate models leverage existing health care delivery and financing structures to accelerate DTx adoption without requiring radical system changes. By leading in this area, local governments exemplify how regional initiatives can maximize DTx potential, establish models for nationwide adoption, and enhance health care delivery through efficient and cost-effective resource allocation across China.

### Limitations

Our analysis faced several key constraints. First, we relied solely on publicly available information, which precluded consideration of cutting-edge research in development, planned clinical trials, and emerging regulations from governments and institutions. Additionally, our focus on published studies, clinical trials, and commercially available products meant we inevitably overlooked insights that might have been gleaned from failures. Finally, the limited amount of real-world evidence for each product restricted our ability to incorporate existing literature on verifying effectiveness in practical settings. Nonetheless, the significance of real-world evidence for DTx cannot be overstated, particularly in the context of health technology assessments that inform reimbursement decisions.

DTx hold great promise for chronic, ophthalmic, psychiatric, and neurological conditions that have traditionally been difficult to manage with conventional methods. Consequently, demand for DTx is expected to rise significantly. However, to move beyond the introductory phase and become a mainstay in health care, DTx must address several challenges. Key among these is the need for robust verification through real-world evidence. Strengthening the integration of research, development, clinical trials, and regulatory frameworks is vital to overcoming this challenge. Additionally, addressing patient engagement barriers and streamlining regulatory processes will be crucial for widespread adoption. The infrastructure and adaptability of DTx within clinical settings must also be carefully considered to ensure stable integration.

China’s experience in advancing DTx provides valuable lessons for other countries. The country’s rapid adoption of digital health solutions, along with its evolving regulatory environment and health care infrastructure, offers insights into how DTx can be scaled effectively. Global stakeholders can draw on these lessons to streamline their own DTx research, development, and implementation, facilitating a faster and more efficient integration of these therapies into health care systems worldwide.

## Appendix

Multimedia Appendix 1 Research data.

Multimedia Appendix 2 PRISMA checklist.

## Abbreviations

AI: artificial intelligence
DiGA: Digitale Gesundheitsanwendungen
DTx: digital therapeutics
HbA_1c_: hemoglobin A_1c_
NMPA: National Medical Products Administration
PRISMA: Preferred Reporting Items for Systematic Reviews and Meta-Analyses
RCT: randomized controlled trial
SMD: standardized mean difference
