# Supplementary material for: Digital Therapeutics in China: Comprehensive Review
Source: J Med Internet Res. 2025 May 27;27:e70955. doi: 10.2196/70955 (PMC12152435; doi:10.2196/70955)
Supplement: Multimedia Appendix 1 [file jmir_v27i1e70955_app1.docx]

Figure S1. The risk of bias analysis by using the Rob-2: adherence by RCT study (detailed judgments for each domain). (A) Meta-analysis of cognitive disorders/dysfunction studies. (B) Meta-analysis of diabetes studies.


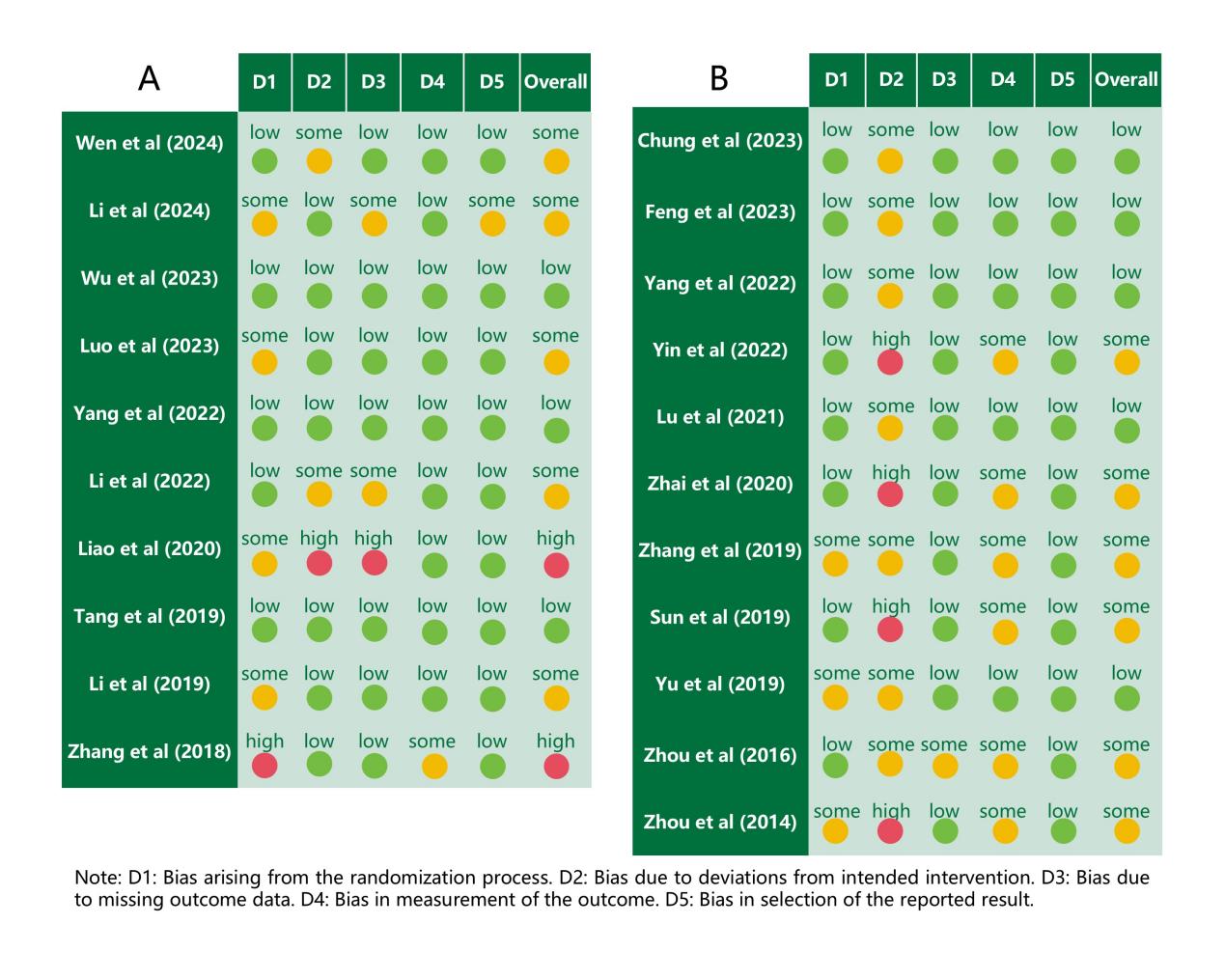


Methods for Meta-Analyses

*Search Strategies and Selection Criteria*

In this systematic review and meta-analysis, we searched PubMed, Embase, Web of Science, IEEE, and Science Direct for publication from database inception up to June 30, 2024, limiting our search to Chinese and English literature. Detailed information of the selection process for meta-analyses and the search terms for each database are provided in the Supplementary appendix. Reference lists of relevant systematic reviews and meta-analyses identified from the database search were screened for potentially eligible studies. Pertinent literature was manually added. Two investigators (XY and NJ) independently screened the titles and abstracts of papers identified from the database search. Papers deemed eligible by both investigators underwent full-text review for final inclusion. Any disagreements throughout the screening process were resolved through discussion, with persistent disagreements adjudicated by a third independent investigator (CH). We used Endnote 20 (Clarivate Analytics, Philadelphia, PA, USA) for study storage and screening, and Microsoft Excel (Microsoft, Redmond, WA, USA) for data extraction and organization.

The inclusion criteria and search strategies were based on population, intervention, comparison, and outcomes (PICO) framework. Studies were eligible for inclusion if they included clinical trials investigating digital therapeutic (DTx) interventions conducted in China. The intervention could involve digital health applications, smartphone-based therapeutic solutions, or other DTx technologies designed for healthcare delivery. Eligible studies had to be randomized controlled trials (RCT) that had a controlled group that received usual care or conventional care.

Studies were excluded if they were non-research articles (such as conference abstracts, editorials, or viewpoints papers), case reports, or observational studies.

This systematic review and meta-analysis is registered at PROSPERO (CRD42024611857 for diabetes and CRD42024615584 for cognitive disorders/dysfunction) followed the PRISMA guidelines.

*Outcomes*

The primary outcome of interest was change in HbA1c levels (%) for diabetes, and change in global cognitive ability for cognitive disorders/dysfunction. HbA1c level was measured via biochemical analyzer. Global cognitive function was measured via standardized neuropsychological tests such as Mini-Mental State Examination (MMSE) and Montreal Cognitive Assessment (MoCA).

*Data analysis*

Study characteristics including study design, population characteristics, intervention characteristics, intervention period, comparison, and outcome measurements. The data was independently extracted and verified by the same two investigators, with disputes resolved as previously described. To calculate effect size, information regarding the sample size and mean (SD) of outcome measure at baseline and post-intervention were collected for individual study. For results not presented as mean (SD), validated methods were used to convert them to the desired format.

Risk of bias was assessed using the Cochrane Risk of Bias 2 (RoB 2) for RCT, which classifies studies into three categories, which are low risk of bias, some concerns, or high risk of bias. The same investigator team independently assessed risk of bias following the previously described resolution process.

The meta-analysis was performed using R (Version 4.3.2), with the “meta” package. A random-effects model was used to calculate the standardized mean differences (SMD) using mean changes from baseline to post-intervention timepoint, the SD at the post-intervention timepoint, and sample size. Positive effect sizes indicated a positive effect of enhancing cognitive function or reducing HbA1c.

To examine heterogeneity beyond sampling error, Higgins I² statistic and Cochran Q statistic (where p < 0.05 indicates heterogeneity) were used. I² values with 95% confidence intervals (95% CI) were categorized as low (25%), moderate (50%), and high (75%) heterogeneity.

Table S1. Search strategies related to clinical research and trials of DTx.

| Databases | Search Strings |
| --- | --- |
| PubMed | (Digital therapeutics OR DTx) AND (digital health OR healthcare OR telemedicine OR telerehabilitation OR mHealth OR eHealth OR Wearable OR Mobile OR Remote OR Smart OR Intelligent OR Wireless OR SaMD OR “smartphone application” OR “smartphone apps” OR WeChat OR “online platform”)) AND (China OR Chinese OR “Hong Kong” OR Macau OR Taiwan OR Taipei) NOT (Review[Publication Type] OR Meta-Analysis[Publication Type] OR Systematic Review[Publication Type])) AND (clinicaltrial[Filter] OR randomizedcontrolledtrial[Filter]) |
| Web of Science | TS=(“Digital therapeutics” OR DTx)  AND  TS=(“digital health” OR healthcare OR telemedicine OR telerehabilitation OR mHealth OR eHealth OR Wearable OR Mobile OR Remote OR Smart OR Intelligent OR Wireless OR SaMD OR “smartphone application” OR “smartphone apps” OR WeChat OR “online platform”) AND  CU=(China OR Chinese OR “Hong Kong” OR Macau OR Taiwan OR Taipei) AND  DT=(Article OR Clinical Trial) NOT  DT=(Review OR “Systematic Review” OR “Meta-Analysis”) |
| IEEE | (((“Digital therapeutics” OR DTx) AND (“digital health” OR healthcare OR telemedicine OR telerehabilitation OR mHealth OR eHealth OR Wearable OR Mobile OR Remote OR Smart OR Intelligent OR Wireless OR SaMD OR “smartphone application” OR “smartphone apps” OR WeChat OR “online platform”) AND (China OR Chinese OR “Hong Kong” OR Macau OR Taiwan OR Taipei)) NOT (“Review” OR “Survey” OR “Systematic Review” OR “Meta-Analysis”)) |
| Science Direct | Find articles with these terms: (“Digital therapeutics” OR DTx) AND (China OR Chinese OR “Hong Kong” OR Macau OR Taiwan) Title, abstract or author-specified keywords: (“digital health” OR healthcare OR telemedicine OR mHealth OR “smartphone application”) Title: NOT (Review OR “Meta-Analysis”) Article type: Research articles |
| Google Scholar | (“Digital therapeutics” OR DTx) AND (“digital health” OR healthcare OR telemedicine OR telerehabilitation OR mHealth OR eHealth OR Wearable OR Mobile OR Remote OR Smart OR Intelligent OR Wireless OR SaMD OR “smartphone application” OR “smartphone apps” OR WeChat OR “online platform”) AND (China OR Chinese OR “Hong Kong” OR Macau OR Taiwan OR Taipei) -Review -“Systematic Review” -“Meta-Analysis” |

Table S2. Major indications of DTx.

| Category | Indications |
| --- | --- |
| Chronic Diseases | Diabetes, Chronic kidney disease, Chronic liver disease, Cardiovascular diseases (CVD), Hypertension, Respiratory conditions, Chronic pain |
| Psychiatric/Mental Health | Psychiatric/Mental disorders (adults), Psychiatric/Mental disorders in Children, ADHD in Children, ASD in Children |
| Cognitive and Neurological | Cognitive disorders/dysfunction (adults), Cognitive disorders/dysfunction in Children, Cranial nerve disorders |
| Substance Use and Addiction | Drug abuse, Smoking cessation, Alcohol use |
| Sleep Disorders | Insomnia, Sleep Apnea, Hypersomnia |
| Cancer | All kinds of cancer and malignant tumors |
| Musculoskeletal and Motor | Musculoskeletal disease, Motor dysfunction/Movement disorders (including swallowing disorders) |
| Obstetrics and Gynecology | Obstetrics and gynecology |
| Infectious Diseases | HIV |
| Sensory Systems | Ophthalmic diseases, Hearing disorders |
| Pediatrics | ADHD in Children, ASD in Children, Cognitive disorders/dysfunction in Children, Psychiatric/Mental disorders in Children |
| Rehabilitation | Motor rehabilitation/Physical therapy, Speech and language rehabilitation |
| Lifestyle and Prevention | Healthy lifestyle promotion, Vaccination, Dosage calculation |
| Other Specific Conditions | Urinary incontinence, Skin conditions, Malnutrition, Injury |

Table S3. Characteristics of the included studies in the meta-analysis of diabetes studies.

| Study | Province | Study design | Population | Clinical setting | Intervention | Comparison | Duration | Follow up | Primary outcome | Second outcome |
| --- | --- | --- | --- | --- | --- | --- | --- | --- | --- | --- |
| Chung et al ^[1]^ (2023) | Taiwan | Open-label, parallel, prospective RCT. | Adults (≥20 years) diagnosed with prediabetes: HbA1c: 5.7%-6.4% or  Fasting plasma glucose: 100-125 mg/dL. Those with cardiopulmonary disease, cancer, other major diseases, or recent use of certain medications were excluded. | Health examination center and outpatient clinics at a teaching hospital in northern Taiwan. | Received usual care plus standard mobile health app. The app included modules for health diary, health education, milestone tracking, and chatroom features. | Received usual care only (15-20 minutes of health education by physicians). | 12 weeks | 1 month | Blood glucose control including Fasting Plasma Glucose (FPG) and Glycated Hemoglobin A1c (HbA_1c_), Body constitution, Body energy, Health-related quality of life (HRQOL). | BMI, Dietary behavior. Physical activity. |
| Feng et al ^[2]^ (2023) | Shanghai | Open-label, parallel, prospective RCT. | Adults (18-79 years) diagnosed with T2DM (HbA1c ≥7%) for ≥6 months. Those who had other serious illnesses, were pregnant or planning pregnancy, were unable to complete follow-up, were unwilling to provide consent, were currently in another study, or had used hypoglycemic agents, β-blockers, thiazide diuretics, nicotinic acid, or steroids within the past 3 months were excluded. | Two community health service centers in Jiading District, Shanghai. | eHealth family-based intervention via WeChat platform plus usual care. Patients had a family member who could use WeChat and lived with or visited them weekly. | Received usual care only (standard community health center management). | 12 months | Follow-up assessment at end of intervention (12 months). | HbA_1c_ level | Self-care activities (diet, exercise, blood sugar testing, foot care, smoking), Risk perception (risk knowledge, personal control, worry, optimism bias, personal risk), Family support (supportive and non-supportive behaviors) |
| Yang et al ^[3]^ (2022) | Chongqing | Open-label, parallel, prospective RCT. | Adults (40-60 years) diagnosed with T2DM (HbA1c: 6.5%-7.5%) and specific blood pressure ranges within past 3 months. Those who had other serious illnesses, eating disorders, psychological disorders, were pregnant/planning pregnancy, were smokers/alcoholics, or participating in other trials were included. | Xinqiao Hospital and Yuzhou road community health service center in Jiulongpo district, Chongqing. | Mobile phone-based telemedicine management via WeChat including: daily uploads of meals, weight, glucose, blood pressure, medications, automated feedback on dietary choices, monthly 40-minute phone consultations, educational content on diet and exercise. | Received routine outpatient care with standard dietary advice and exercise recommendation. | 12 months | Clinical examinations at baseline and 12 months. | Blood glucose measurement (FPG and HbA_1c_), Body composition parameters, Blood pressure, Quality of life (SF-36), Weight, BMI, waist circumference. | Lipid profiles (TG, TCH, LDL-c, HDL-c), Total treatment costs for 1 month |
| Yin et al ^[4]^ (2022) | Jiangsu | Open-label, parallel, prospective RCT. | Adults (18-55 years) diagnosed with T2DM (HbA1c: 7%-10%) for >6 months. Those who had serious cardiovascular disease, pregnancy, obesity surgery history, COVID-19 infection, or other major comorbidities were excluded. | Outpatient endocrine  Clinic at the First People’s Hospital of Xuzhou, Jiangsu | Hospital’s telemedicine app for blood glucose monitoring offered: custom-tailored dietary recommendations, exercise guidance, weekly medical advice and monitoring. | Received conventional outpatient clinic appointments and usual care. | Initial 21-day home isolation period and  6-month intervention period. | Assessments at baseline, 22 days (after isolation), 3 months, and 6 months. | Blood glucose control (FPG and HbA_1c_), SDS (Self-Rating Depression Scale) scores. | Body composition parameters, Blood pressure, Lipid profiles, BMI, Waist-to-hip ratio. |
| Lu et al ^[5]^ (2021) | Tianjin | Open-label, parallel, prospective RCT. | Adults (18-75 years) diagnosed with T2DM (HbA1c: 7%-10%) who were on medication adjustment. Those who were pregnant or breastfeeding, had deafness or mental disabilities that could interfere with proper follow-up care, or were diagnosed with serious conditions were excluded. | Inpatient department of Integrated Traditional Chinese and Western at one Tianjin Hospital. | WeChat-based medication consultation and guidance. Medication alarm clock and instruction services. Regular online follow-up. Blood glucose data transmission via Bluetooth. | Standard care per hospital procedures: Outpatient visits every 2 weeks, Self blood glucose monitoring at home. | 6 months | The intervention group had weekly follow-ups in month one, bi-weekly in months 2-3, and monthly thereafter, while the control group attended bi-weekly clinic visits. Both completed a 6-month follow-up. | Change in HbA_1c_ level and in proportion of patients achieving HbA1c <7%. | FPG, Daily medication cost, Number of medication types, Hypoglycemic events. |
| Zhai et al ^[6]^ (2020) | Tianjin | Open-label, parallel, prospective RCT. | Adults (18-60 years) diagnosed with T2DM for ≥3 months. Those who had serious complications, cognitive impairment, type 1 diabetes, pregnancy, malignancy, or were participating in other studies were included. | Community Health Service Center of Zhangjiawo Town, Xiqing District, Tianjin. | Diabetes management app "YuTangYiHu" with connected glucometer for blood glucose monitoring, giving diet advice and medication guidance. Patients received online instruction from education nurse. | Received conventional diabetic treatment only. | 6 months | Assessments at baseline, 3 months, and 6 months. | HbA_1c_ level | Diabetes Self-Efficacy Scale (DSES) scores measuring: Self-efficiency on diet, Regular exercise, Medication, Blood glucose monitoring, Foot care, Prevention and management of high/low blood glucose. |
| Zhang et al ^[7]^ (2019) | Shanghai | Open-label, parallel, prospective RCT. | Adults (18-65 years) diagnosed with poorly controlled diabetes for ≥6 months, HbA1c ≥8% within 3 months before enrollment. Those who were insulin pump users, pregnant or planning pregnancy during the study period, excessive drinkers or drug users, those who used drugs affecting blood sugar in the previous 3 months, patients receiving treatment for psychotic conditions, those with severe complications or systemic diseases, patients who experienced cardio- or cerebrovascular events in the previous 6 months, individuals with severe hearing or visual impairment were excluded. | The outpatient clinic of Shanghai Jiao Tong University Affiliated Sixth People’s Hospital, Department of Endocrinology and Metabolism, Shanghai. | Self-management app (Welltang app) provided: Educational content about diabetes management, Self-management tools for recording blood glucose, diet, exercise, medication, and weight, Patient community features, Communication channel with clinicians. | Received usual care without app installation. Patients learned diabetes knowledge through self-learning and adopted lifestyle changes voluntarily. | 6 months | Assessments at baseline, 3 months, and 6 months. | HbA_1c_ level | FPG, Body weight, Lipid levels (triglycerides, HDL-c, LDL-c), Blood glucose test rate, Frequency of app usage, Guiding time. |
| Sun et al ^[8]^ (2019) |  | Open-label, parallel, prospective RCT. | Adults (＞65 years) with T2DM (HbA1c: 7%-10%), without illiteracy, abnormal liver and kidney function, severe diabetic  complications, use of insulin pumps, and participation in other  clinical trials. | Department of Endocrinology of one affiliated hospital in Jilin. | mHealth management system with glucometer data transmission via bluetooth. Medical team review and advice every 2 weeks via messaging/phone. Diet management software for daily dietary records. | Standard care through conventional outpatient clinic appointments. Regular self-monitoring without additional tracking. | 6 months | Regular outpatient clinic visits at 3-month intervals. Physical examinations and blood tests at baseline, 3 months, and 6 months. | HbA_1c_ level | Postprandial blood glucose levels, Blood biochemical indices, Patient compliance (frequency of uploading blood glucose data), Patient satisfaction (7-point questionnaire). |
| Yu et al ^[9]^ (2019) | Shanghai | Open-label, parallel, prospective RCT. | Adults (35-65 years) with T2DM (No HbA_1c_ limitation defined), without severe liver/kidney diseases, insulin pump use, pregnancy, mental illness, visual impairment, or inability to access web. | The outpatient  department at one Shanghai hospital. | Mobile phone application (MPA) “Diabetes-Carer” app offered diabetes education, self-management, patient community, and real-time communication with clinicians. | No MPA or self-monitoring of blood glucose. | 24 weeks | Assessments at baseline, week 12, and week 24. | Change in HbA_1c_ level and in proportion of patients achieving HbA1c <7%. | FPG and 1,5-anhydroglucitol levels. |
| Zhou et al ^[10]^ (2016) | Zhejiang | Open-label, parallel, prospective RCT. | Adults (18-74 years) diagnosed with diabetes (No HbA_1c_ limitation defined), without severe complications. | The outpatient department of endocrinology at the one affiliated hospital in Zhejiang. | Smartphone-based Welltang diabetes management app offer: Knowledge database on diet, exercise, medicine guidelines; Self-management tools for blood glucose, diet tracking; Communication with clinicians;  Standard care. | Received usual standard care only. | 3 months | Assessments at baseline and 3 months. | HbA_1c_ level | Blood glucose (fasting and 2h post-breakfast), LDL cholesterol, Weight, Blood pressure, Hypoglycemic events, Patient satisfaction with app use, Diabetes knowledge, Self-care behaviors. |
| Zhou et al ^[11]^ (2014) | Guangdong | Open-label, parallel, prospective RCT. |  | The outpatient  department at one affiliated hospital in Guangzhou, Guangdong. | A telemedicine system to upload patients’ blood glucose and other metabolic information at home at least every 2 weeks. The researchers then provided appropriate advice based on their key behaviors. | Received traditional face-to-face visits without any specific telemedicine intervention. | 3 months | Follow-up assessment at end of intervention (3 months). | HbA_1c_ level | FBG, incidence of hypoglycemia, and achievement of HbA1c target <7%. |

Table S4. Characteristics of the included studies in the meta-analysis of cognitive disorders/dysfunction studies.

| Study | Province | Study design | Population | Clinical setting | Intervention | Comparison | Duration | Follow up | Primary outcome | Second outcome |
| --- | --- | --- | --- | --- | --- | --- | --- | --- | --- | --- |
| Wen et al ^[12]^  (2024) | Jilin | Open-label, parallel, prospective RCT. | Urban adults (≥45 years) residents diagnosed with Mild Cognitive Impairment (MCI) per 2018 Chinese Guidelines, financially able to afford cognitive training, without prior systematic cognitive training in past year, without severe physical diseases or mental disorders, without long-term psychotropic drug use, without severe anxiety (HAMA ≥14) or depression (HAMD ≥17). | Outpatient of memory clinic of a grade-A hospital in Changchun, Jilin. | Standard nursing care. Home-based occupational therapy (OT). Computer-assisted cognitive training (CCT). | Standard nursing care only (including paper-pencil cognitive training). | 12 weeks | 3 months | Cognitive function measured by Mini-Mental State Examination (MMSE) and Montreal Cognitive Assessment (MoCA). | Anxiety (HAMA scale), Depression (HAMD scale), Activities of daily living (ADL scale). |
| Li et al ^[13]^ (2024) | Beijing | Open-label, parallel, prospective RCT. | Patients with Vascular Cognitive Impairment No Dementia (VCIND), diagnosed with cognitive impairment without dementia and small vessel ischemia. | Department of Neurology and Department of Radiology at Xuanwu Hospital in Beijing. | CCT including: Processing speed, attention, perception, Long-term and working memory, Calculation, executive control, reasoning, Problem-solving, 30-minute daily sessions with adaptive difficulty levels. | Fixed cognitive training focused only on: Processing speed, Attention, Fixed primary difficulty level throughout. | 7 weeks | Follow-up at  Baseline, week 7 (post-intervention) and month 6 (follow-up). | Changes in functional connectivity (FC) of brain networks assessed by fMRI. | Cognitive performance measured by:  MoCA for global cognition, Trail Making Test B-A (TMT B-A) for executive function, Boston Naming Test (BNT) for linguistic function. |
| Wu et al ^[14]^ (2023) | Fujian | Single-blind, parallel, prospective RCT. | MCI right-handed  patients (50-85 years) with MoCA scores ≤25 points, being at stage 2-3 on Global Deterioration Scale, without contraindications for MRI. | Rehabilitation departments from three hospitals affiliated with Fujian Traditional Chinese Medicine University. | 24 one-hour CCT sessions over 8 weeks. Three sessions per week using FDA approved Cognitive Assessment and Rehabilitation Training Machine. Multi-domain training (attention, memory, processing speed etc.). | Standard care only. Instructed to refrain from cognitive training. | 8 weeks | Post-intervention assessment within 1 week after completion and 3 month follow-up. | Global cognitive function measured by MoCA. | Processing speed (DSST), Cognitive flexibility (Stroop), Episodic memory (CAVLT), Nonverbal memory (Rey CFT), Brain functional connectivity changes measured by MRI. |
| Luo et al ^[15]^ (2023) | Fujian | Single-blind, parallel, prospective RCT. | MCI right-handed  Patients (>60 years) who were diagnosed with MCI by Peterson’s criteria, with MMSE score ≥17 for illiterate, >20 for 1-6 years education, >24 for ≥7 years education. | Memory Clinic of Fujian Provincial Hospital and Community Health Service Center of Gulou District, Fuzhou | 60-minute remote expressive arts program (rEAP) sessions delivered via electronic devices with WeChat platform. Two sessions per week for 12 weeks. Included visual art creation and storytelling activities. | Health education sessions twice weekly for 12 weeks delivered by geriatric nurses. Focus on cognitive health maintenance and prevention strategies. | 12 weeks | Post-intervention assessment within 2 weeks after completion. | Global cognitive function measured by MoCA. | DSST, Stroop, Memory function (AVLT), Executive function (Shape Trail Test)  Language proficiency (VFT, BNT), Brain functional connectivity measured by fMRI. |
| Yang et al ^[16]^ (2022) | Guangdong | Open-label, parallel, prospective RCT. | Community-dwelling adults (aged ≥65 years) diagnosed with MCI, with over 95% lived with spouse or children. | Two large regional communities in Guangzhou. | Dietary guidance (face-to-face meetings every 3-4 weeks). Physical training (progressive muscle strength and aerobic exercise). 60-90 min CCT weekly sessions. Management and monitoring of metabolic indicators and vascular risk factors. | Usual care including three 45-min health education classes during the 6-month period covering epidemiology, etiology, clinical manifestations, prognosis of MCI, and recommendations for social activities. | 6 months | Data collected at baseline, 1 month, 3 months, and 6 months. | Global cognitive function measured by MoCA. Comprehensive physical capacity (Short Physical Performance Battery and Timed Up and Go test). Depression (15-item Geriatric Depression Scale). Quality of Life in Alzheimer’s Disease scale). | Not explicitly specified. |
| Li et al ^[17]^  (2022) | Hong Kong | Open-label, parallel, prospective RCT. | Community-dwelling adults with MCI who were diagnosed according to National Institute on Aging-Alzheimer’s Association criteria. | Community centers of three non governmental organizations in Hong Kong. | BRAVE program with three components:  Mobile application providing brain health info and exercise videos  Peer volunteer training program  Group-based multicomponent exercise intervention (3 sessions/week for 8 weeks)  Group format (8-10 participants per group) with peer volunteer support | Usual care including social and leisure activities. No structured exercise or cognitive training activities. | 8 weeks | Assessments at baseline, immediately post-intervention, and 3 months post-intervention. | Cognitive function measured by Alzheimer’s Disease, Assessment Scale-Cognitive subscale (ADAS-Cog), Colour Trails Test (CTT), Digit Span Forward and Backward tests. | HRQoL measured by SF-36. Participant satisfaction survey. Program engagement metrics (app logins, video views). |
| Liao et al ^[18]^  (2020) | Taiwan | Single-blind, parallel, prospective RCT. | Community-dwelling adults (aged ≥65 years) diagnosed with MCI, with MMSE score ≥24 and MoCA score <26. | Communities and day care centers in Taipei. | VR-based physical and cognitive training. Using VIVE system with VR glasses and motion controllers  IADL-based functional tasks in VR (shopping, food preparation, etc.). | Combined physical and cognitive training. Resistance exercises, aerobic exercise, balance exercises. Simultaneous cognitive training during physical exercises. | 12 weeks | Assessed at baseline and post-intervention. No additional follow-up after intervention completion. | MoCA, Executive function (EXIT-25). Verbal memory (CVVLT). Brain activation measured by NIRS during cognitive tasks. Instrumental activities of daily living (IADL). | Not explicitly specified. |
| Tang et al ^[19]^  (2019) | Beijing | Open-label, parallel, prospective RCT. | Patients with subcortical VCIND. | Three medical centers in Beijing. | Multidomain, adaptive CCT including processing speed, attention, orientation, memory, calculation, etc. | Waitlist control receiving usual care. | 7 weeks | Follow-up at baseline, immediately post-intervention, and 3 months post-intervention. | Global cognitive function measured by MoCA. Executive function measured by TMT B-A. | Brain functional connectivity changes. Brain structural changes. Quality of life. |
| Li et al ^[20]^ (2019) |  | Open-label, parallel, prospective RCT. | MCI patients diagnosed based on CDR score=0.5 and medial temporal lobe atrophy, with Chinese Han descent with >6 years of education. Screening included MMSE, anxiety and depression scales. | Memory clinics in Shanghai. | CCT program with 8 tasks: Visual working memory, 30-second memory, Episodic memory, Speed of calculation, Visual search, Alertness, Mental rotation, Image re-arrangement. | Received only observation and follow-up. | 6 months | Follow-up in initial 6-month intervention period. Additional 12-month follow-up period after intervention (total 18 months). | Changes in MMSE scores. | Neuropsychological tests including Addenbrooke’s cognitive examination-revised (ACER), Auditory verbal learning test, Shape trail test, Complex figure test  Symbol digit substitution test, Stroop Color-Word Test. fMRI measures of brain activity. |
| Zhang et al ^[21]^ (2018) | Jilin | Open-label, parallel, prospective non-RCT with control. | Elderly MCI patients (aged ≥60 years) diagnosed based on Petersen’s criteria, with preserved general intellectual functioning (MMSE), memory impairment on MoCA-BJ, intact daily living activities, and were willing to participate. | Long-term care facilities in Changchun. | MESSAGE communication strategy combined with group reminiscence therapy (GRT). Used multimedia elements including electronic albums, videos, and music. | Receiving no intervention. | 12 weeks | Assessments at Baseline, 6 weeks, and 12 weeks (end of intervention). | Cognitive function measured by MMSE and MoCA-BJ. Quality of life measured by Chinese (mainland) version of SF-36. | Individual cognitive domains from MoCA-BJ including: Visuospatial/executive function, Naming, Attention, Language, Abstract thinking, Delayed memory, Orientation. |

References

1. Chung, H. W., Tai, C. J., Chang, P., Su, W. L., & Chien, L. Y. (2023). The Effectiveness of a Traditional Chinese Medicine-Based Mobile Health App for Individuals With Prediabetes: Randomized Controlled Trial. JMIR mHealth and uHealth, 11, e41099. <https://doi.org/10.2196/41099>
2. Feng, Y., Zhao, Y., Mao, L., Gu, M., Yuan, H., Lu, J., Zhang, Q., Zhao, Q., & Li, X. (2023). The Effectiveness of an eHealth Family-Based Intervention Program in Patients With Uncontrolled Type 2 Diabetes Mellitus (T2DM) in the Community Via WeChat: Randomized Controlled Trial. JMIR mHealth and uHealth, 11, e40420. <https://doi.org/10.2196/40420>
3. Yang, L., Xu, J., Kang, C., Bai, Q., Wang, X., Du, S., Zhu, W., & Wang, J. (2022). Effects of Mobile Phone-Based Telemedicine Management in Patients With Type 2 Diabetes Mellitus: A Randomized Clinical Trial. The American journal of the medical sciences, 363(3), 224–231. <https://doi.org/10.1016/j.amjms.2021.09.001>
4. Yin, W., Liu, Y., Hu, H., Sun, J., Liu, Y., & Wang, Z. (2022). Telemedicine management of type 2 diabetes mellitus in obese and overweight young and middle-aged patients during COVID-19 outbreak: A single-center, prospective, randomized control study. PloS one, 17(9), e0275251. <https://doi.org/10.1371/journal.pone.0275251>
5. Lu, Z., Li, Y., He, Y., Zhai, Y., Wu, J., Wang, J., & Zhao, Z. (2021). Internet-Based Medication Management Services Improve Glycated Hemoglobin Levels in Patients with Type 2 Diabetes. Telemedicine journal and e-health : the official journal of the American Telemedicine Association, 27(6), 686–693. <https://doi.org/10.1089/tmj.2020.0123>
6. Zhai, Y., & Yu, W. (2020). A Mobile App for Diabetes Management: Impact on Self-Efficacy Among Patients with Type 2 Diabetes at a Community Hospital. Medical science monitor : international medical journal of experimental and clinical research, 26, e926719. <https://doi.org/10.12659/MSM.926719>
7. Zhang, L., He, X., Shen, Y., Yu, H., Pan, J., Zhu, W., Zhou, J., & Bao, Y. (2019). Effectiveness of Smartphone App-Based Interactive Management on Glycemic Control in Chinese Patients With Poorly Controlled Diabetes: Randomized Controlled Trial. Journal of medical Internet research, 21(12), e15401. <https://doi.org/10.2196/15401>
8. Sun, C., Sun, L., Xi, S., Zhang, H., Wang, H., Feng, Y., Deng, Y., Wang, H., Xiao, X., Wang, G., Gao, Y., & Wang, G. (2019). Mobile Phone-Based Telemedicine Practice in Older Chinese Patients with Type 2 Diabetes Mellitus: Randomized Controlled Trial. JMIR mHealth and uHealth, 7(1), e10664. <https://doi.org/10.2196/10664>
9. Yu, Y., Yan, Q., Li, H., Li, H., Wang, L., Wang, H., Zhang, Y., Xu, L., Tang, Z., Yan, X., Chen, Y., He, H., Chen, J., & Feng, B. (2019). Effects of mobile phone application combined with or without self-monitoring of blood glucose on glycemic control in patients with diabetes: A randomized controlled trial. Journal of diabetes investigation, 10(5), 1365–1371. <https://doi.org/10.1111/jdi.13031>
10. Zhou, W., Chen, M., Yuan, J., & Sun, Y. (2016). Welltang - A smart phone-based diabetes management application - Improves blood glucose control in Chinese people with diabetes. Diabetes research and clinical practice, 116, 105–110. <https://doi.org/10.1016/j.diabres.2016.03.018>
11. Zhou, P., Xu, L., Liu, X., Huang, J., Xu, W., & Chen, W. (2014). Web-based telemedicine for management of type 2 diabetes through glucose uploads: a randomized controlled trial. International journal of clinical and experimental pathology, 7(12), 8848–8854.
12. Wen, X., Song, S., Tian, H., Cui, H., Zhang, L., Sun, Y., Li, M., & Wang, Y. (2024). Intervention of computer-assisted cognitive training combined with occupational therapy in people with mild cognitive impairment: a randomized controlled trial. Frontiers in aging neuroscience, 16, 1384318. <https://doi.org/10.3389/fnagi.2024.1384318>
13. Li, Q. G., Xing, Y., Zhu, Z. D., Fei, X. L., Tang, Y., & Lu, J. (2024). Effects of computerized cognitive training on functional brain networks in patients with vascular cognitive impairment and no dementia. CNS neuroscience & therapeutics, 30(6), e14779. https://doi.org/10.1111/cns.14779
14. Wu, J., He, Y., Liang, S., Liu, Z., Huang, J., Liu, W., Tao, J., Chen, L., Chan, C. C. H., & Lee, T. M. C. (2023). Effects of computerized cognitive training on structure‒function coupling and topology of multiple brain networks in people with mild cognitive impairment: a randomized controlled trial. Alzheimer's research & therapy, 15(1), 158. <https://doi.org/10.1186/s13195-023-01292-9>
15. Luo, Y., Lin, R., Yan, Y., Su, J., Lin, S., Ma, M., & Li, H. (2023). Effects of Remote Expressive Arts Program in Older Adults with Mild Cognitive Impairment: A Randomized Controlled Trial. Journal of Alzheimer's disease : JAD, 91(2), 815–831. <https://doi.org/10.3233/JAD-215685>
16. Yang, Q. H., Lyu, X., Lin, Q. R., Wang, Z. W., Tang, L., Zhao, Y., & Lyu, Q. Y. (2022). Effects of a multicomponent intervention to slow mild cognitive impairment progression: A randomized controlled trial. International journal of nursing studies, 125, 104110. <https://doi.org/10.1016/j.ijnurstu.2021.104110>
17. Polly W C Li, Doris S F Yu, Parco M Siu, Schwinger C K Wong, Bernice S Chan, Peer-supported exercise intervention for persons with mild cognitive impairment: a waitlist randomised controlled trial (the BRAin Vitality Enhancement trial), Age and Ageing, Volume 51, Issue 10, October 2022, afac213, <https://doi.org/10.1093/ageing/afac213>
18. Liao, Y. Y., Tseng, H. Y., Lin, Y. J., Wang, C. J., & Hsu, W. C. (2020). Using virtual reality-based training to improve cognitive function, instrumental activities of daily living and neural efficiency in older adults with mild cognitive impairment. European journal of physical and rehabilitation medicine, 56(1), 47–57. <https://doi.org/10.23736/S1973-9087.19.05899-4>
19. Tang, Y., Xing, Y., Zhu, Z., He, Y., Li, F., Yang, J., Liu, Q., Li, F., Teipel, S. J., Zhao, G., & Jia, J. (2019). The effects of 7-week cognitive training in patients with vascular cognitive impairment, no dementia (the Cog-VACCINE study): A randomized controlled trial. Alzheimer's & dementia : the journal of the Alzheimer's Association, 15(5), 605–614. <https://doi.org/10.1016/j.jalz.2019.01.009>
20. Li, B. Y., He, N. Y., Qiao, Y., Xu, H. M., Lu, Y. Z., Cui, P. J., Ling, H. W., Yan, F. H., Tang, H. D., & Chen, S. D. (2019). Computerized cognitive training for Chinese mild cognitive impairment patients: A neuropsychological and fMRI study. NeuroImage. Clinical, 22, 101691. <https://doi.org/10.1016/j.nicl.2019.101691>
21. Zhang, H. H., Liu, P. C., Ying, J., Shi, Y., Wang, S. Q., Zhang, M. L., & Sun, J. (2018). Evaluation of MESSAGE communication strategy combined with group reminiscence therapy on elders with mild cognitive impairment in long-term care facilities. International journal of geriatric psychiatry, 33(4), 613–622. <https://doi.org/10.1002/gps.4822>
